# Challenging the point neuron dogma: FS basket cells as 2-stage nonlinear integrators

**DOI:** 10.1038/s41467-019-11537-7

**Published:** 2019-08-14

**Authors:** Alexandra Tzilivaki, George Kastellakis, Panayiota Poirazi

**Affiliations:** 1 Institute of Molecular Biology and Biotechnology (IMBB), Foundation for Research and Technology Hellas (FORTH), Heraklion, 70013 Greece; 2 Department of Biology, University of Crete, Heraklion, 70013 Greece; 3 Present Address: Einstein Center for Neurosciences Berlin, Charité – Universitätsmedizin Berlin, Freie Universität Berlin and Humboldt-Universität zu Berlin, and Berlin Institute of Health, NeuroCure Cluster of Excellence, Charitéplatz 1, 10117 Berlin, Germany

**Keywords:** Long-term memory, Biophysical models, Network models, Learning algorithms

## Abstract

Interneurons are critical for the proper functioning of neural circuits. While often morphologically complex, their dendrites have been ignored for decades, treating them as linear point neurons. Exciting new findings reveal complex, non-linear dendritic computations that call for a new theory of interneuron arithmetic. Using detailed biophysical models, we predict that dendrites of FS basket cells in both hippocampus and prefrontal cortex come in two flavors: supralinear, supporting local sodium spikes within large-volume branches and sublinear, in small-volume branches. Synaptic activation of varying sets of these dendrites leads to somatic firing variability that cannot be fully explained by the point neuron reduction. Instead, a 2-stage artificial neural network (ANN), with sub- and supralinear hidden nodes, captures most of the variance. Reduced neuronal circuit modeling suggest that this bi-modal, 2-stage integration in FS basket cells confers substantial resource savings in memory encoding as well as the linking of memories across time.

## Abstract (web summary)

Recent experimental work has revealed non-linear dendritic integration in interneurons. Here, the authors show, through detailed biophysical modeling, that fast spiking interneurons are better described with a 2-stage artificial neural network model calling into question the use of point neuron models.

## Introduction

GABAergic interneurons play a key role in modulating neuronal activity and transmission in multiple brain regions^1–4^. Among others, they are responsible for controlling the excitability of excitatory and inhibitory cells, modulating synaptic plasticity, and coordinating synchrony during neuronal oscillations^3,5–7^. GABAergic interneurons come in a variety of molecular profiles, anatomical features, and electrophysiological properties^2,8–10^. Despite this variability, many interneuron types exhibit similar computations, the most common being a precise EPSP-spike coupling^11–13^. As they innervate a large number of cells, near the site of action potential initiation, they are believed to generate a powerful widespread inhibition, also referred to as an inhibitory blanket^14^.

Fast Spiking basket cells (FS BCs) constitute one of the main types of hippocampal and neocortical interneurons^3,9,13,14^. They are part of the PV positive interneuron class, which also includes the axo-axonic, chandelier, and bistratified subtypes^3^. FS BCs are distinguished from other subtypes by their anatomical features, synaptic connectivity patterns, and membrane mechanisms^12–15^. These include the presence of calcium permeable AMPA (cp-AMPA) receptors^14,16–18^, the low expression of NMDA receptors^19,20^, a weak backpropagation of APs^14,20^, a low density of sodium channels^14^, and a high density of potassium channels in their aspiny dendritic trees^13,14,20^.

A growing body of the literature recognizes the importance of FS BCs in controlling executive functions, such as working memory and attention as well as their role in neurodegenerative disorders^14,21–23^. However, little is known about the mechanistic underpinnings of FS BC contributions to these functions. Most studies have focused on the molecular and anatomical features of FS BCs^7,13^ and led to the dogma that FS BCs serve as “on–off” cells, integrating inputs like linear—or at best sublinear—point neurons^13,14,24^.

This dogma is based on the assumption that FS BCs integrate synaptic inputs in a linear manner, completely ignoring potential dendritic influence. Dendritic integrative properties however, can play a pivotal role in translating incoming information into output signals^25,26^. In pyramidal neurons for example, this is often done in highly nonlinear ways that facilitate memory and other executive functions^27–29^.

Exciting new findings suggest a potentially similar contribution of dendrites in interneuron function. Sublinear dendritic EPSP integration along with supralinear calcium accumulations has been reported in cerebellar stellate cells^11,30^. Moreover, certain interneuron subtypes in the CA1 area exhibit dendritic supralinearities^7,31^ while in the CA3, both calcium nonlinearities and sodium spikes in FS BC dendrites during sharp wave ripples have been reported^7^. The exact nature of dendritic computations in FS BCs, however, is unknown. As a result, whether a linear point neuron or a more sophisticated abstraction—like the two-stage^32^ or multistage integration proposed for pyramidal neurons—can successfully capture their synaptic integration profile, remains an open question.

**Fig. 1:**
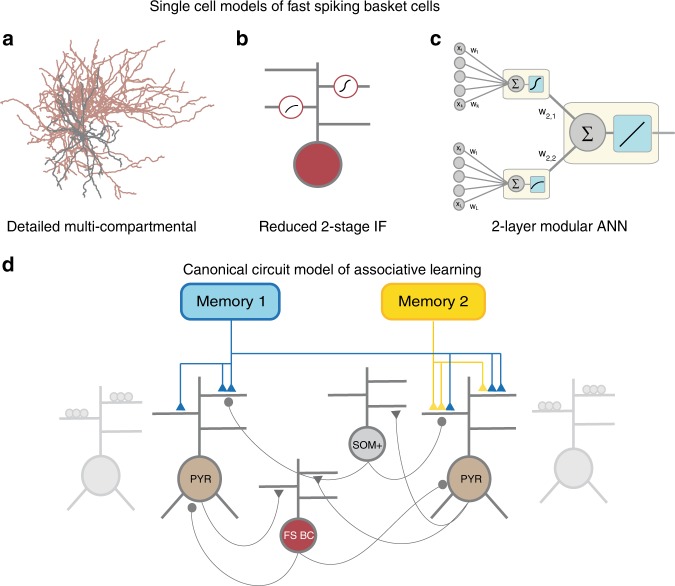
Modeling tools used to study dendritic integration in FS BCs and its functional implications. **a** Detailed, biophysically constrained multi-compartmental models using realistic anatomical reconstructions. **b** Reduced two-stage integrate and fire models of FS BCs. **c** two-layer ANN reduction describing the FS BCs. **d** Reduced network model with simplified pyramidal, FS BCs and SOM + interneurons. FS BCs and SOM+ interneurons provide feedback inhibition to excitatory neurons, with FS BCs targeting the somatic subunit while SOM+ neurons target the dendritic subunits. Memory encoding afferents provide inputs to excitatory cell dendrites

**Fig. 2:**
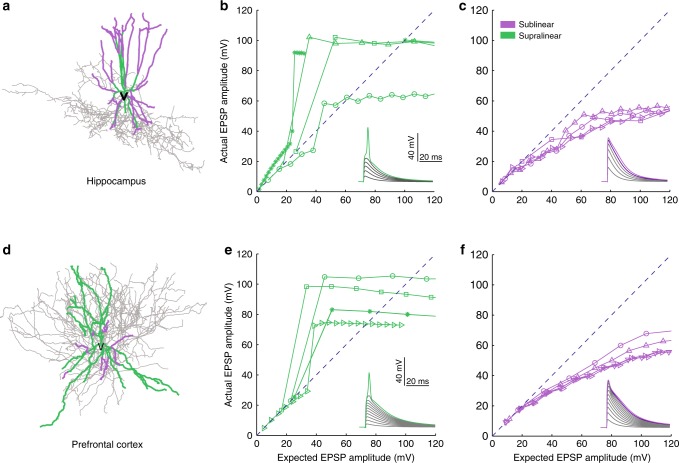
Bimodal dendritic integration in multi-compartmental FS BC models. Examples of hippocampal (**a**) and PFC (**d**) FS BC morphological reconstructions. Representative input-output curves from supralinear (**b**, **e**) and sublinear (**c**, **f**) dendritic branches in hippocampal (top) and PFC (bottom) models, in response to synaptic stimulation. Increasing numbers of synapses (from 1 to 20 with step = 1) are uniformly distributed within each stimulated branch and are activated with a single pulse. The *y*-axis shows the amplitude of the dendritic EPSP caused by synaptic activation while the *x*-axis shows the expected EPSP amplitude that would result from the linear summation of synaptic EPSPs. The dashed line indicates linear summation. Insets show representative traces

**Fig. 3:**
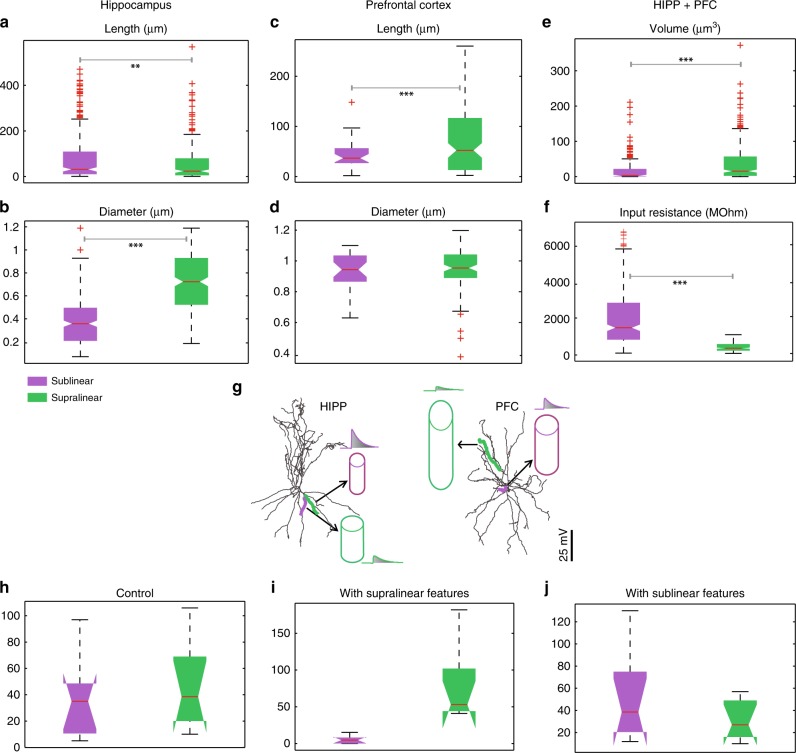
Morphological determinants of dendritic integration mode. **a**, **c** Total length distributions of supralinear vs. sublinear dendrites in the hippocampus (**a**) and the PFC (**c**). Statistically significant differences are observed for both sub- and supra-linear dendrites, in both areas. (*p*-value < 0.0001 for hippocampus and *p*-value < 0.01 for PFC, Student's *t*-test). **b**, **d** Same as in **a**, **b**, for mean dendritic diameter. Statistically significant differences are observed in hippocampal (*p*-value < 0.0001 Student’s *t*-test) but not in PFC FS BCs. **e**, **f**. Dendritic Volume and dendritic Input resistance are common discriminating characteristics among supralinear (larger, with low input resistance) and sublinear (smaller, with high input resistance) dendrites, for both areas (*p*-value < 0.0001 Student’s *t*-test for hippocampus and PFC, for Volume and Input resistance respectively). **g** Schematic illustration of morphological features for supralinear and sublinear dendrites in hippocampus (left) and PFC (right). Traces indicate the first EPSP in supralinear and sublinear dendrites. **h**–**j** Distributions of the number of supralinear and sublinear dendrites in both areas, under control conditions (**h**), with the mean diameter and length of all dendrites set to the mean values of the supralinear class (**i**) and with mean diameter and length of all dendrites set to the mean values of the sublinear class (**j**). Error bars indicate the minimum and maximum values

**Fig. 4:**
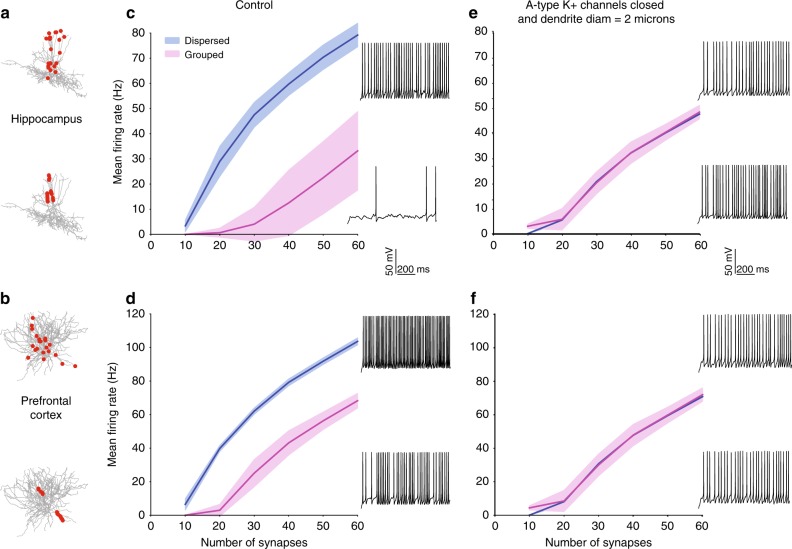
Effect of bimodal dendritic integration on neuronal firing. Firing rate responses (in Hz) from one hippocampal (**a**,**c**) and one PFC (**b**,**d**) model cell, in response to stimulation of increasing numbers of synapses (10–60) that are either randomly distributed throughout the entire dendritic tree (blue) or grouped within a few dendritic branches (pink). Synapses are stimulated with a 50 Hz Poisson spike train. In both cases, dispersed activation leads to higher firing rates. **e**, **f** Same as in **c**, **d** with dendritic diameter set to 2 μm and removal of A-type dendritic channels. Firing rates are indistinguishable between grouped and dispersed activation patterns. Insets depict representative traces from dispersed (top) and grouped (bottom) activation of 30 synapses. Red dots in **a**, **b**, show the synaptic allocation motif

**Fig. 5:**
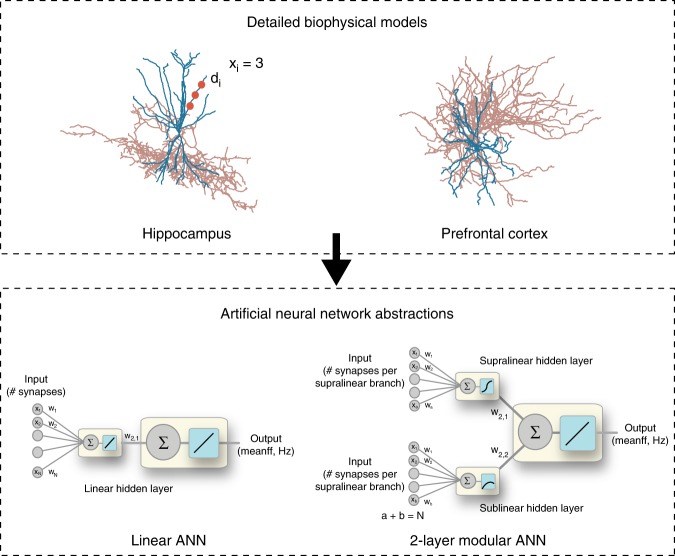
Reduction of multi-compartmental models into ANN abstractions. Two types of abstractions are examined: (a) a Linear ANN, in which the input from all dendrites (*x*_i_ = number of synapses in dendrite i, *N* = number of dendrites) is linearly combined at the cell body and (b) a two-layer modular ANN, in which the input is fed into two parallel, separated hidden layers. The supralinear-layer receives the number of inputs landing onto supralinear branches (*a* = number of supralinear dendrites) while the sublinear layer receives the number of inputs landing onto sublinear dendrites (*b* = number of sublinear dendrites). Neurons in both hidden layers are equipped with nonlinear transfer functions, a logistic sigmoid in the supralinear layer and a sublinear function in the sublinear layer. The somatic transfer functions of both ANNs are linear

**Fig. 6:**
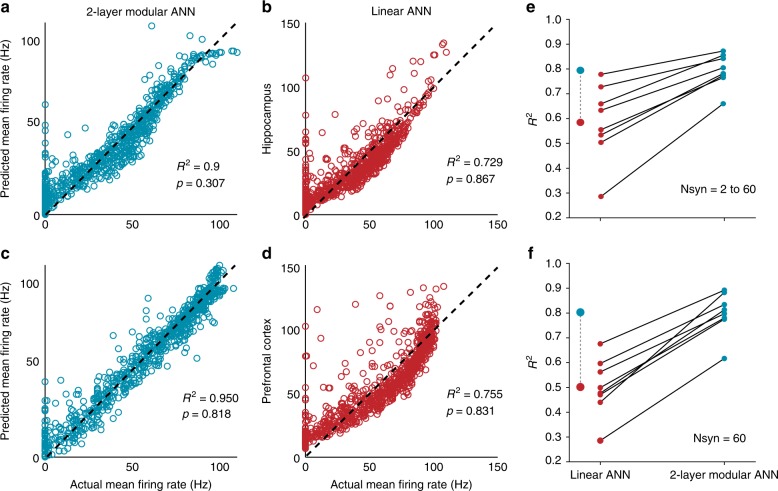
Challenging the point neuron dogma: FS basket cells as two-stage nonlinear integrators. Linear regression analysis for two-layer modular (**a**, **c**) and linear (**b**, **d**) ANNs for one indicative hippocampal (top) and one indicative PFC (bottom) model cell. Actual mean firing rates (Hz) correspond to the responses of the compartmental model when stimulating with 50 Hz Poisson spike trains varying numbers of synapses (1–60), distributed in several ways (grouped or dispersed) within both sub- and supra-linear dendrites. Expected mean firing rates (Hz) are those produced by the respective ANN abstraction when receiving the same input (number of stimulated synapses) in its respective sub-/supra- or linear input layer nodes. **e** Regression performance (measured as *R*^2^) for two-layer modular (right) and Linear (left) ANNs for all eight FS BC model cells, respectively. In all cases the two-layer modular ANN is superior to the Linear ANN. Mean *R*^2^ values over all cells for the Linear (red) and two-layer modular (cyan) ANNs are shown on the left. **f** Same as **e**, applied to datasets comprised of 60 input synapses. The difference in performance between the two ANN types is higher in this challenging task

**Fig. 7:**
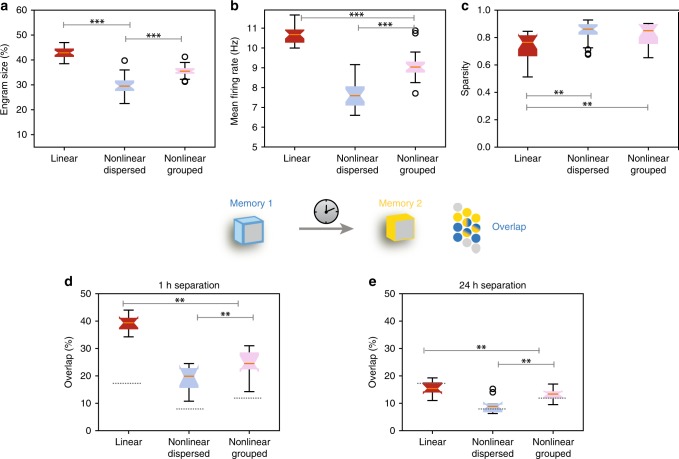
Properties of memory engram encoding under different dendritic nonlinearity configurations. **a** Size of memory engram (percentage of excitatory neurons that respond with *ff* > 10 Hz during memory recall) for Linear/Bi-modal FS-BC dendritic subunits receiving dispersed (light blue) or grouped (pink) synaptic inputs. **b** Mean firing rate of the excitatory population under the conditions enumerated in **a**. **c** Treves–Rolls sparsity metric of the excitatory population firing rates under the conditions enumerated in **a**. **d** Percentage of overlap between two memory engrams when two memories are separated by 1 h, under the conditions enumerated in **b**. Dashed lines indicate the chance level of overlap for the engram sizes of the dispersed case shown in **a**. **e** As in **d** for 24 h separation. Box plots indicate data from 20 simulation trials for **a**–**c**, and 10 trials for **d**–**e**. ANOVA **p < 0.05, ***p < 0.005. Error bars indicate minimum and maximum values

To address these questions, we developed an elaborate toolset that consists of (a) detailed, biologically constrained biophysical models of hippocampal and cortical FS BCs, (b) reduced two-stage integrate-and-fire models of these cells, (c) two-layer artificial neural network abstractions and (d) a large microcircuit model of two-stage pyramidal, FS BC and dendrite targeting (SOM) interneurons (See “Methods” and Fig. 1). We first characterized the integration profiles of FS BC dendrites using the detailed biophysical models. Synaptic stimulation predicted the co-existence of two distinct modes within the same tree: some dendrites exhibited supralinear while others sublinear summation of inputs (Fig. 2, Supplementary Figs. 4 and 5). Supralinear dendrites supported local, sodium-dependent spikes (Supplementary Fig. 7) and were characterized by large volume and low input resistance (Fig. 3), which are shaped by the combination of dendritic length and diameter. Direct manipulation of these anatomical features in biophysical models gated the induction of sodium spikes and determined the integration mode (Fig. 3). Using an array of different activation patterns, we found that spatially dispersed inputs lead to higher firing rates than inputs which are grouped within a few dendrites (Fig. 4), opposite to respective findings in pyramidal neurons^33^. Moreover, these different activation patterns result in a wide range of firing rates that are better explained by a two-layer artificial neural network (ANN) with nonlinear hidden layer activation functions rather than a linear ANN (Figs. 5, 6, Table 1). Finally, in order to assess the functional implications of these predictions, we built a reduced network model of two-stage integrator neurons (Fig. 1d) and showed that bi-modal nonlinear integration in FS BCs is beneficial for memory engram storage as well as the linking of memories across time (Fig. 7).

**Table 1:** ANN regression performance (*R*^2^) for individual sets of synapses in the eight model cells

ANN type	20 Synapses	40 Synapses	60 Synapses
Two-layer modular	0.8224	0.7432	0.6167
ANN	0.8228	0.906	0.8339
	0.8048	0.8709	0.7971
	0.7951	0.8563	0.8127
	0.8242	0.8352	0.7752
	0.9098	0.8857	0.8921
	0.8879	0.8975	0.8827
	0.8415	0.86	0.7801
Linear ANN	0.4541	0.3484	0.2856
	0.6814	0.7462	0.5966
	0.6436	0.5201	0.5625
	0.5636	0.4475	0.4768
	0.555	0.4919	0.4707
	0.7832	0.7242	0.6758
	0.6263	0.5463	0.44
	0.6179	0.7039	0.4991

Comparison of ANN prediction accuracy (measured as the *R*^2^) for linear and two-layer modular ANN reductions across all eight FS BC models, tested on three sets of synaptic inputs consisting of 20, 40, or 60 activated synapses, respectively. Synapses were randomly distributed in various ways/locations in the biophysical model cells and resulting firing rates were used as target vectors for the ANNs. The two-layer modular ANN is clearly superior to the Linear ANN when it comes to capturing location-induced firing-rate variability

This work provides a systematic, cross-area analysis of dendritic integration in FS BCs and its functional implications. Our findings challenge the current dogma, whereby interneurons are treated as linear summing devices, essentially void of dendrites. We predict that the dendrites of FS BCs in both hippocampal and neocortical regions can operate in distinct nonlinear modes. As a result, FS BCs, similar to pyramidal neurons^32^, are better represented by a two-stage integrator abstraction rather than a point neuron. Importantly, nonlinear dendritic integration in these cells offers substantial advantages for memory encoding in largescale networks.

## Results

### Multi-compartmental, biophysical models

A total of eight biophysical model neurons were built using realistic reconstructions of FS BCs from rat hippocampal areas (CA3, 5 cells)^33^ and from the prefrontal cortex of mice (three cells)^34^ (Supplementary Fig. 5). To ensure biological relevance, ionic and synaptic conductances as well as basic membrane properties of model cells were heavily validated against experimental data^12–15,35–37^ (Supplementary Tables 1–4, Supplementary Figs. 1–3). Moreover, for consistency reasons, the same set of biophysical mechanisms (type and distribution) was used in all model cells.

### Bi-modal dendritic integration in Fast Spiking basket cells

The first step for deducing a realistic abstraction of FS BCs is the systematic characterization of dendritic/neuronal integration properties across a significant number of neurons and dendrites. Towards this goal, we simulated gradually increasing excitatory synaptic input to the dendrites of all neuronal models and recorded the voltage response both locally and at the soma^11,32^. Increasing numbers of synapses (1–20) were uniformly distributed in each stimulated dendrite and activated synchronously with a single pulse. For this particular experiment, sodium conductances in somatic and axonal compartments were closed to avoid backpropagation contamination effects^38,39^ that were detectable in some dendrites. We compared measured EPSPs to their linearly expected values, given by the number of activated inputs multiplied by the unitary EPSP. We found that within the same dendritic tree, branches summate inputs either in a supralinear or a sublinear mode (Fig. 2, Supplementary Figs. 4, 5). While there were differences in the number of dendrites and proportions of sub- vs. supralinear dendrites, all of the morphologies tested expressed both integration modes (Supplementary Table 5). Moreover, while both modes have been suggested in distinct interneuron types^11,31,40^, their co-existence in the same tree has yet to be reported.

To assess the robustness of this finding, we first performed a sensitivity analysis whereby the cp-AMPA, NMDA, VGCCs, sodium, and A-type potassium conductances were varied by ±20% of their control value. We found no changes in the integration mode of dendrites and only insignificant alterations in the spike threshold of supralinear dendrites (Supplementary Fig. 6b). The only manipulation that eliminated supralinearity was the blockade of dendritic sodium channels (Supplementary Fig. 7).

Next, we examined whether the two modes are influenced by the presence of gap junctions, which are well established in FS BCs^14,41^. Towards this goal, we connected pairs of hippocampal and PFC cells with 10 electrical synapses (see “Methods”). Presynaptic cells were synaptically activated so as to fire at gamma rate frequency as per Tamas et al.^41^ and the integration mode was assessed, as previously, in the dendrites of the postsynaptic cell. We found no influence of gap junctions on the integration mode, apart from a slightly increased membrane potential (Supplementary Fig. 8).

The same effect was observed in simulations of more physiological conditions such as active whisking^42^. This was done via weak synaptic activation of randomly selected dendrites resulting in a somatic firing rate of 3 ± 1 Hz^42^ (see “Methods”). Both modes of dendritic integration remained unaffected by the presence of in vivo like activity fluctuations (Supplementary Fig. 9).

Taken together, the above simulations establish the robustness of bi-modal dendritic integration and suggest that under physiological conditions, FS BCs are likely to express both types of dendritic integration modes.

### Determinants of dendritic integration modes

Next, we searched for biophysical and/or anatomical determinants of the two integration modes. Blockade of sodium conductances in the dendrites eliminated the supralinear integration mode in all morphologies tested (Supplementary Fig. 7), but this was not the case for blockade of cp-AMPA, NMDA, VGCCs or A-type potassium channels (Supplementary Fig. 6a). These simulations indicate that sodium channels are the key ionic mechanism underlying the supralinear mode. What remains unclear is why these model cells also have sublinear dendrites, when the distribution and conductance values of sodium channels are the same in all dendrites.

Since morphological features of dendrites were previously shown to influence synaptic integration profiles^43^, we investigated whether anatomical features correlate with the expression of each integration mode. We found that the mean dendritic diameter was highly statistically different (*p*-value = 2.6041e−60) among sub-(thinner) and supra-linear (thicker) dendrites in the hippocampus (Fig. 3b), while in the PFC the dendritic length was a better determinant of sub- (shorter) vs. supra-linearity (longer) (*p*-value = 4.1768e−04) (Fig. 3c). Length was less, yet, important in the hippocampus (*p*-value = 0.0040) (Fig. 3a) while diameter was not different among sub- and supralinear dendrites in the PFC (*p*-value = 0.9458) (Fig. 3d). Dendritic volume and input resistance consider both of the above anatomical features and serve as robust morphological/electrophysiological determinants for all dendrites in both areas (*p*-value = 9.8516e−11, 3.9457e−45, respectively), (Fig. 3e, f).

Overall, we found that supralinear dendrites have high volume and low input resistance while sublinear dendrites have smaller volume and high input resistance (Fig. 3e, f). This can be explained by considering the fast kinetics of cp-AMPA receptors and A-type potassium channels in the dendrites of FS BCs. In sublinear dendrites, where the input resistance is high (small volume), coincident synaptic input induces a large, fast rising EPSP which in turn strongly activates the A-type potassium channels that rapidly repolarize the membrane, thus preventing the branch from spiking^39^. The opposite is true for supralinear dendrites, where the low input resistance results is smaller depolarizations that drive smaller A-type potassium currents, enabling the branch to reach the sodium spike threshold. This explanation is consistent with prior findings^14,39^.

To test the above proposition, we performed causal manipulations whereby we fixed the diameter and length of all dendrites to the mean values of first the supralinear and then the sublinear class and assessed the effect on integration mode. We found that setting the dendritic anatomy to that of a given class also dictated the integration mode (Fig. 3h–j). These findings suggest that, under the experimentally constrained conductance values for sodium channels, morphology plays a crucial role in the ability of a given dendrite to support local sodium spikes and express the supralinear integration mode.

### Effect of bimodal dendritic integration on neuronal firing

To assess the impact of bimodal dendritic integration on neuronal output, we simulated a large variety of different spatial patterns of synaptic activation and measured the resulting firing rates. Specifically, we generated over 10,000 synaptic stimulus patterns, which comprised of increasing numbers of excitatory synapses. Synapses were either placed within a few, strongly activated branches (grouped) or they were randomly distributed within the entire dendritic tree (dispersed). In all cases, synapses were activated with random Poisson spike trains at 50 Hz (see “Methods”). Dendrites were selected at random and inputs were distributed uniformly within selected dendrites. For the dispersed case, we allocated 2, 5, or 10 synapses in randomly selected dendrites, one at a time, while for the grouped case we allocated 10, 15, 20, 30, and 60 synapses within an increasing number of branches. In all cases, the number of activated synapses increased gradually up to a maximum of 60, as this number was sufficient to induce spiking at gamma frequencies (30–100 Hz). This process was repeated *k* times (*k* = number of dendrites in each cell) to ensure full coverage of the entire tree. As expected given the two modes of dendritic integration, the localization of activated inputs affected neuronal firing. For a given number of activated synapses, dispersed activation led to higher somatic firing rates than grouped activation, particularly during gamma related frequencies (30–100 Hz) both in hippocampal (Fig. 4c) as well as in PFC FS basket cells (Fig. 4d). Interestingly, this finding is opposite to what has been reported for pyramidal neurons, in which synapse clustering increases firing rates^32,44^.

It was previously proposed that the combination of a small diameter with an increased conductance of A-type potassium channels in FS BCs underlies the preference for dispersed synaptic allocations^14^. To tests this hypothesis, we repeated the above experiment after increasing the diameter (to 2 μm) and blocking the A-type potassium conductance in all dendrites. As shown in Fig. 4e, f, this manipulation resulted in very similar firing rates irrespectively of the spatial arrangement of synapses, thus eliminating the preference for dispersed allocation of excitatory inputs.

Supplementary Fig. 12 shows the relative contributions of these two mechanisms in our model cells. Disperse synaptic arrangements benefit mostly from the dendritic morphology of FS BCs, as setting the dendritic diameter to 2 μm sharply decreases this preference (Supplementary Fig. 12a, b). This is likely because small diameters prevent signal loss, enabling the small depolarizations produced by dispersed inputs to reach and excite the soma. Grouped arrangements on the other hand, are severely hampered by the high conductance of the A-type potassium channels^14,39^, as blockade of these currents enhances somatic output (Supplementary Fig. 12c, d). This is because grouped—but not disperse—inputs induce large dendritic depolarizations which strongly activate A-type channels. Since NMDA currents, which would further boost and prolong the induced EPSPs, are very small in these neurons^19^, the hyperpolarizing effects of the A-type currents are larger than the depolarizing effects of grouped activation.

Another factor that contributes to disperse preference is dendritic integration. Unlike pyramidal neurons where dendrites are mostly supralinear and benefit from grouped inputs via the induction of dendritic spikes^27,28,32^, these neurons also have sublinear dendrites which dampen the abovementioned benefit. The higher the percentage of sublinear dendrites, the larger the dampening, as: (1) the probability of allocating inputs in the few supralinear dendritic branches is much smaller and (2) activating sublinear dendrites with grouped inputs offers little/no advantage as dendritic spikes do not occur in these branches. As shown in Supplementary Table 7, the more sublinear dendrites a FS BC model has, the weaker the response to the more restricted, grouped input.

Taken together, this analysis reveals that the combination of a high conductance of A-type channels (which penalizes grouped inputs), the specific morphological features of FS BCs (which favor dispersed inputs), and the presence of multiple sublinear dendrites underlie the preference of these model cells for disperse rather than grouped activation of their inputs, contrary to pyramidal neuron models^32^.

### FS basket cells as two-layer artificial neural networks

The nonlinear synaptic integration taking place within the dendrites of cortical^45^ and CA1^28,29^ pyramidal neurons was previously described as a sigmoidal transfer function^32^. Based on this reduction, a single pyramidal neuron was proposed to integrate its synaptic inputs like a two-layer artificial neural network, where dendrites provide the hidden layer and the soma/axon the output layer. To assess whether a similar mathematical formalism could be ascribed to FS BC models, we constructed linear and nonlinear artificial neural networks (as graphically illustrated in Fig. 5) and asked which of them can better capture the firing rate variability in the biophysical models.

Specifically, four types of feedforward, artificial neural networks (ANNs) were constructed (see “Methods”). In the two-layer modular ANN, supralinear, and sublinear dendrites were simulated as two parallel hidden layers consisting of a logistic sigmoid and a sublinear (*y*(*x*) *=* (*x* + 2)^0.7^−2) activation function, respectively (Fig. 5). The number of activated synapses allocated to supralinear vs. sublinear dendrites in the biophysical model,s was used as input to the respective hidden layers. The output layer represented the soma/axon of the biophysical model and consisted of a linear activation function. In the linear ANN, there was only a single hidden layer receiving input from all dendrites and consisting of linear activation functions (Fig. 5). We also constructed two ANNs with the exact same architecture as the linear one, but with either (a) a logistic sigmoidal (two-layer supralinear ANN) or (b) a sublinear *y*(*x*) *=* (*x* + 2)^0.7^ *−* 2, (two-layer sublinear ANN) activation function in the hidden layer neurons (Supplementary Fig. 10). The latter ANNs represent FS BCs with just one type of nonlinear dendrites.

For all eight FS BC model neurons the linear and two-layer modular ANNs were trained using the number of synapses to supra-/sublinear dendrites as inputs to the respective hidden layers and the mean firing rate of the soma as target output. A randomly selected 80% of our synaptic activation data set was used to train the model and the rest 20% to test its generalization performance (see “Methods”). Performance accuracy was estimated based on regression analysis between the ANN-generated firing rates and those produced by the biophysical models. Fits for two representative model cells are shown in Fig. 6a–d, while the overall performance for all eight model cells is shown in Fig. 6e. Fig. 6f demonstrates the performance of both ANN types for a dataset of the same power (number of inputs = 60), whereby the location of the inputs varies. As evident from the results, the two-layer modular ANN outperformed the linear ANN in all cases tested.

However, the performance of the linear ANN was relatively good. This can be attributed to the wide range of activated synapses (2–60) which resulted in large differences in the somatic firing, irrespectively of synapse location, and can thus be captured by any linear model (similar findings were seen in pyramidal model cells in Poirazi et al. ^32^). Therefore, we also assessed the performance accuracy of linear and two-layer modular ANNs to the more challenging task of discriminating between input distributions corresponding to the exact same number of synapses. To do so, we subdivided the data into input categories corresponding to 20, 40, and 60 synapses, respectively. In these more challenging conditions, the two-layer modular ANN clearly outperformed the respective linear ANN, which failed to explain the variance produced by differences in input location. This result was consistent for all model cells as shown in Table 1. Performance for the 60-synapse case is shown in Fig. 6f.

Taken together, this analysis suggests that a two-layer artificial neural network that considers both types of dendritic nonlinearities is a much better mathematical abstraction for FS basket cells than the currently assumed linear point neuron.

### Bimodal nonlinear integration of FS basket cells enhances memory encoding

In order to investigate the functional implications of our findings, we adapted a previously developed canonical microcircuit model^46,47^, to incorporate simplified two-stage excitatory neurons, FS BCs and dendrite targeting (SOM+) interneurons (Fig. 1d and Supplementary Table 6). The model includes inhibitory feedback connectivity, multi-dendrite and perisomatic targeting interneurons. It implements plasticity-related processes which act on multiple temporal and spatial scales: calcium-dependent LTP/LTD with synaptic tagging and capture (STC), NMDA dendritic spike plateaus, plasticity of intrinsic neuronal excitability and homeostasis (see “Methods”). The modeling of memory engrams is based on fear memory studies in the hippocampus^48,49^. The encoding of memories in the neuronal population takes place after the concurrent pairing of stimulation of afferents encoding an unconditioned stimulus (e.g., contextual information) simultaneously with the conditioned stimulus (e.g., shock). We evaluated the size of memory engrams via the percentage of excitatory neurons which were strongly activated during the recall of the memory (upon presentation of the conditioned stimulus alone), 24 h after encoding. The size of the memory engram for the control condition was calibrated according to experimental data for the CA1 area^49,50^.

The network model was first trained to encode a single memory^46^ (see “Methods”) using FS BCs with either (a) purely linear or (b) bimodal (sublinear and supralinear) dendritic subunits, as predicted by the compartmental modeling analysis (Fig. 2). SOM + interneurons were modeled as having either sublinear, linear, supralinear, or bimodal dendritic subunits (Supplementary Fig. 11). In these simulations, synaptic inputs to the FS BCs cells were either a) randomly distributed in all dendrites (dispersed) or b) grouped in a few branches (~33%) of all dendrites (grouped) (see “Methods”). The properties of the resulting memory engram (*i.e*., the population of active excitatory neurons activated by presentation of the unconditioned stimulus) were assessed by analyzing the activity of excitatory neurons during recall 24 h after the learning event (Fig. 7e).

Our results indicate that, compared to linear dendrites, bimodal FS BC dendrites lead to significant reductions in the size of the resulting memory engram (*p*-value = 5.8e−15), and the mean engram firing rates (*p*-value = 3.1e-18 for linear-dispersed, p-value = 7.2e−10 for linear-grouped) (Fig. 7a, b) while they also increase the network firing sparsity (*p*-value = 0.00095 for linear-dispersed, *p*-value = 0.00338 for linear-grouped) (Fig. 7c). All of the above suggest that dendritic bimodality in FS BCs promotes resource savings in the encoding of new memories.

Summarizing, the memory engram properties indicate that bi-modal FS BC dendrites receiving dispersed inputs confer resource consumption advantages to memory encoding by a) increasing the sparsity of the population, b) recruiting fewer engram neurons and c) reducing the overall network excitability. The above findings were unaffected by the presence of either linear, supralinear or bi-modal SOM + model dendrites (Supplementary Fig. 11).

Finally, we also assessed the role of FS-BC nonlinearities in memory linking, by encoding two memories separated by 1 or 24 h in the same network model and measuring the population overlap of the resulting memory engrams. According to previous work^44,46,49^, memories learned in close temporal proximity (e.g., 1 h apart) display increased engram overlap compared to distant memories (24 h apart). Overlapping storage is also associated with behavioral binding of the two memories and has been proposed to underlie the linking of memories across time^49^. We found that linear FS BC dendrites result in substantially larger engram overlaps in the circuit model compared to bi-modal dendrites (Fig. 7d), for the 1-h case. These overlaps are in fact significantly larger than the experimentally reported ones^49^ (which are about ~20%), suggesting that the two memories may interfere with one another. Taken together, our network modeling analysis suggests a beneficial role of nonlinear dendrites in FS BCs with respect to memory encoding, storage capacity as well as the binding of memories over time.

## Discussion

The role of dendrites in interneuron computations is a rapidly emerging and debatable subject^14,40^. Several recent reports present exciting findings according to which dendrites may serve as key players^7,11,14,30,31,40,51^. For example, sodium spikes and supralinear calcium accumulation have recently been reported in the dendrites of FS BCs^7^, yet the consensus still favors the linear point neuron dogma^13,14^. The present study provides new insight into this ongoing debate by systematically analyzing the dendritic integration mode of FS BCs in two brain areas: The Hippocampus and the PFC. We do so, using an extensive set of computational tools that extends from detailed biophysical single cell models, to reduced integrate-and-fire single cell and circuit models as well as artificial neural network models (Fig. 1). We predict that dendrites of both cortical and hippocampal FS BCs operate in one of two modes of synaptic integration: supralinear or sublinear (Fig. 2). Supralinearity is due to the generation of dendritic sodium spikes (Supplementary Figs. 7), which are in turn gated by the morphology (Fig. 3) of dendrites. Moreover, we find that somatic output is influenced by the spatial distribution of activated synapses, with dispersed input inducing higher firing rates than grouped activation. This feature is opposite to pyramidal neurons^32,44^ and is attributed to a) the presence of sublinear dendrites in FS BCs and b) the small dendritic diameter^14,52^, increased A-type current and fast EPSP kinetics of cp-AMPA receptors found in these cells^14,39^ (Fig. 4, Supplementary Fig. 12). Due to these properties, a two-layer modular artificial neural network abstraction with both sub- and supra-linear hidden neurons (Fig. 5) captures the spiking profile of biophysical neurons with much higher accuracy than a linear ANN, analogous to a point neuron. This is true for all of the 8 morphological reconstructions of FS BCs tested and is more evident for datasets in which the number of inputs is fixed but their location varies (Fig. 6, Table 1). This is because discriminating the effect of input location as opposed to input strength is a much more challenging task and pushed the linear ANN to its performance limits. Finally, we show that such a two-stage integration model facilitates the efficient encoding, storage and discriminability of memories in a biologically relevant circuit model across time (Fig. 7).

A bimodal dendritic integration is predicted for all hippocampal and PFC morphologies analyzed. Supralinearity was found to be due to the occurrence of dendritic sodium spikes (Supplementary Fig. 7). Several mechanisms can influence the generation of such dendritic spikes: ionic conductances (primarily of sodium currents but also potassium currents) and morphological features. In our models, biophysical mechanisms are constrained by existing experimental data and dendritic sodium conductances are kept to a minimum (10 times smaller than the soma^14^), so as to minimize the probability of non-physiological dendritic spiking. Sensitivity analysis further demonstrates that results are robust to physiological variations in a wide range of active dendritic conductances (Supplementary Fig. 6). These findings strongly suggest that dendritic spiking in certain dendrites of FS basket cells is highly likely to occur under physiological conditions, in line with recent experimental reports^7^.

Apart from sodium currents as a universal enabling mechanism, we find a key role of morphology in gating local dendritic spikes. A combination of dendritic length and mean diameter, or otherwise the dendritic volume and input resistance, is statistically different between sub- (smaller) and supralinear (larger) dendrites across all morphologies tested (Fig. 3). The inability of small-volume dendrites (Fig. 3f) to support sodium spikes is attributed to their high input resistance (Fig. 3f), fast kinetics of calcium permeable AMPA receptors and the high density of A-type potassium channels^14,39^. This combination results in large, fast EPSPs that are very efficient in activating I_A_ currents, which in turn repolarize the membrane^14^. This mechanism has been previously proposed by others^39^, is supported by our morphology and I_A_ manipulation experiments (Fig. 4), and is in line with other studies reporting a similar effect of morphology on the ability of dendrites to generate local spikes^43,53^.

Our simulations predict the co-existence of both sublinear and supralinear dendrites in all FS BCs models (Fig. 2, Supplementary Figs. 4–9). Similar bimodal dendritic integration has been reported in hippocampal CA1 pyramidal neurons^27,28,32,54^ and predicted in PFC pyramidal neurons^45^.

The existence of sublinear dendritic branches supports the idea of inhibitory neurons acting as coincidence detectors by aggregating spatially disperse and nearly synchronous synaptic inputs^14^. Moreover, sublinear dendrites can compute complex nonlinear functions similar to those computed by sigmoidal dendrites^55^, thus substantially extending the processing capacity of these neurons compared to a linear integrator. Why have two types of nonlinearity then?

Our network modeling predicts that the presence of both types of nonlinearities confer substantial benefits to network computations and especially to memory encoding. We find that bimodal nonlinearities in the dendrites of FS BCs, enables the encoding of new memories within a smaller neuronal population, thus increasing sparsity and storage capacity. These nonlinearities also facilitate the interaction of memories over time, via decreasing the possibility of interference (Fig. 7).

Artificial neural network analysis demonstrates that a FS basket cell is better described by a two-stage abstraction that incorporates both modes of dendritic integration (Figs. 5, 6). This work, along the lines of the two-stage model proposed for pyramidal neurons^32^, strongly challenges the prevailing point neuron dogma. The two-stage abstraction is supported by experimental reports of dendritic sodium spikes and supralinear calcium accumulations^7,31^ while it also explains sublinear dendritic integration^13,14,55^, providing a unifying framework for interneuron processing.

Possible limitations of our work include the imprecise modeling of ionic and synaptic mechanisms given the shortage of sufficient information for FS BCs models. This limitation is counteracted by the sensitivity analysis of the mechanisms that mostly influence our findings and their consistency across several cortical and hippocampal morphologies. Another limitation is the lack of inhibitory inputs (except from the autaptic GABAa current that is incorporated in all models). Inhibitory inputs consist of just 6% of all incoming contacts in Fast Spiking interneurons^9,14^. Thus, our results are unlikely to be affected by inhibitory inputs. FS basket cells in the hippocampus and the neocortex are highly interconnected by gap junctions^14,41^, that can speed the EPSP time course, boost the efficacy of distal inputs and increase the average action potential frequency after repetitive synaptic activation^14^. All of these effects would contribute to stronger responses but unless gap junctions are spatially specific to certain branches and not others, they are unlikely to influence the nonlinear integration modes of dendrites. Finally, given the great anatomical and biophysical heterogeneity of different interneurons^2,3^, whether similar dendritic computations are present in other interneuron subtypes will need to be investigated on a case-by-case basis.

## Conclusion

This work provides a novel view of dendritic integration in FS basket cells that extends in hippocampal and cortical areas. Here we suggest new reductionist models for interneuron processing, in which dendrites play a crucial role. Experimental validation of these new models is likely to change the way we think about interneuron processing, attribute new and exciting roles to FS basket cells and open new avenues for understanding interneuron contributions to brain function.

## Methods

### Compartmental modeling

All eight model neurons were implemented within the NEURON simulation environment (version 7.3)^56^. Detailed morphological reconstructions of the five Fast Spiking basket cells of the rat Hippocampus (CA3) were adopted from Tukker et al.^33^, via the NeuroMorpho.org database (Fig. 1). Due to the lack of axonal compartments for some cell reconstructions, we used the axon from the B13a.CNG.swc reconstruction for all five hippocampal neuron models. The three PFC morphologies were adopted from Rotaru et al.^34^, via the NeuroMorpho.org database (Fig. 1) and included their respective axons.

Dendritic branches with mean diameter values larger than 1.2 μm were excluded from all simulations and data analysis procedures, based on Emri et al.^52^. The NLM Morphology Viewer Software was used to transform morphological reconstructions into .hoc files.

### Biophysical properties

All model neurons were calibrated with respect to their biophysical properties so as to conform to experimental data. The same active and passive properties were used in all model cells, with the exception of very small modifications in the conductances of somatic/axonal sodium and delayed rectifier potassium channels (Kdrin) (Supplementary Tables 1 and 2). The latter were necessary to account for the influence of morphological variability on neuronal responses according to experimental evidence^14^.

Conductances of all active ionic mechanisms were adapted from Konstantoudaki et al.^57^. Both hippocampal and PFC models include fast voltage-dependent sodium channels (gnafin), delayed rectifier potassium channels (gkdrin), slow inactivation potassium channels (gslowin), slow calcium dependent potassium channels (gkcain), A-type potassium channels for proximal and distal dendritic regions (gkadin, gkapin), h currents (ghin), and L-, N- and T-type voltage-activated calcium channels (gcal, gcan and gcat, respectively). Sodium current conductances were substantially larger in axonal compared to somatic compartments, which were in turn an order of magnitude larger than dendritic sodium conductances^13^. Moreover, dendritic branches located beyond 100 μm from the soma (distal dendrites) had smaller sodium conductances than proximal branches (located less than 100 μm from the soma) as per^14,39^. A calcium buffering mechanism was included in all compartments. Details about all biophysical mechanisms are listed in Supplementary Table 2.

### Synaptic conductances

Calcium permeable (GluR2-lacking) AMPA, NMDA and autaptic GABAa synaptic currents were incorporated in all model cells. Synaptic weights were validated so as to reproduce the current waveforms depicted in Wang and Gao^19^ and Bacci et al.^58^ and are shown in Supplementary Table 4 and Supplementary Fig. 2.

### Electrophysiological validation

All model neurons were heavily validated against experimental data in order to ensure biological plausibility. Averaged electrophysiological values for the model cells and respective experimental values are shown in Supplementary Table 3.

### Bi-modal dendritic integration in Fast Spiking basket cells

To map the dendritic integration profiles of our model neurons, we activated increasing numbers of synapses (1–20, with step 1) in each dendrite and recorded the voltage responses both locally and at the cell body for 100 ms. Synaptic input was simulated as a single depolarizing pulse, as per Poirazi et al. ^29^. Sodium conductances in somatic and axonal compartments were set to zero in order to avoid backpropagation effects^14,39^. Twelve autaptic events were also activated in somatic compartments^59^.

Integration modes were deduced by comparing the measured dendritic/somatic responses (Actual EPSP amplitude) against what would be expected if synaptic depolarizations summed linearly (Expected EPSP amplitude). A dendrite was termed supralinear if Actual responses were larger than Expected, even if this was true only for a short range of synaptic inputs. A dendrite was considered sublinear if the Actual EPSPs were smaller than the Expected values for all synaptic inputs tested.

Sensitivity analysis was performed by modifying the conductances of NMDA, calcium-permeable AMPA receptors, Voltage gated Calcium Channels (VGCCs), Sodium, and A-type (proximal and distal) mechanisms by ±20%. Increasing numbers of synapses (for 1–40) were used to assess possible changes in single branch integration. Results for all manipulations are shown in Supplementary Fig. 4.

### Modeling of gap junctions

Ten gap junctions (GJs) with 0.4 μS conductance were simulated as shown in Tamas et al.^41^. Two in the soma and eight in randomly selected proximal dendrites, all located in neuron *b* (post synaptic neuron) and originating from ten proximal dendrites of neuron *a* (pre synaptic neuron). The current generated by a gap junction (I_GJ_) was modeled as:

1 $$I_{\mathrm{GJ}} = g_{\mathrm{gap}}(v_{\mathrm{post}} - v_{\mathrm{pre}}),$$

where g_gap_, v_post_, and v_pre_ are the GJ conductance, the post- and the pre-synaptic membrane potentials, respectively, as per Minecci et al.^60^. Neuron *a*, exhibits firing with gamma band frequency of 30–33 Hz, in response to 50Hz poisson spike train stimulation of randomly allocated synapses, Tamas et al.^41^. In Neuron *b*, we simulated the typical, single burst activation of uniformly allocated synapses (1–20, with step 1) in each dendrite respectively, while eliminating 90% of the sodium conductances in axosomatic compartments. This protocol was applied to one connected pair of PFC and one connected pair of hippocampal neurons. Results are representative of the other neuron models.

### Modeling in vivo like background fluctuations

Randomly selected dendrites received sparsely activated synapses (32 excitatory and 8 inhibitory) so as to generate 3 ± 1 Hz firing in soma thus simulating active whisking conditions according to Gentet et al.^61^. The typical single burst activation of uniformly allocated synapses (1–20, with step 1) was applied in each stimulated dendrite. respectively. Simulations were performed under conditions of  90% blockade of sodium conductances in axosomatic compartments. This protocol was applied to one PFC and one hippocampal pair of neurons and is representative to all neuron models.

### Morphological determinants of dendritic integration mode

Mean dendritic diameter and total dendritic length for supralinear and sublinear dendrites were measured for all reconstructed neurons. Dendritic volume was calculated according to the following formula:

2 $$V = \pi \cdot \left(\frac{diam}{2}\right)^2 \cdot length (\mu m^3)$$

Dendritic Input Resistance was calculated according to the following formula:

3 $${{\mathrm{R}}_{\mathrm{in}}} = \frac{\mathrm{DV}}{I}(MOhm),$$

where *I* = −100 pA (4) injected in each dendritic branch and DV is the generated IPSP.

Statistical analysis for all morphological features was performed using Student’s *t*-test with equal sample sizes and assuming unequal variances (Welch’s *t*-test).

### Synaptic stimulation protocols

In all stimulation protocols, inputs were activated using a 50 Hz Poisson spike train. The maximum number of activated synapses was 60, as it was sufficient to induce firing at gamma related frequencies (30–100 Hz). The spatial arrangement of activated synapses was defined according to each of the following stimulation protocols:

### Randomly dispersed synaptic stimulation protocol

Different numbers of synapses (Nsyns = 2–60) were randomly placed on randomly selected dendrites. For a given number of synapses Nsyn, at each allocation step, one dendrite was chosen at random and one synapse was placed at a random location within this dendrite. For the selected dendrite, synaptic location was randomly changed five times. This process was repeated *N* times, where *N* was the number of dendrites for each model cell. This process ensured a random distribution of activated synapses within the entire dendritic tree of each modeled neuron.

### Grouped synaptic stimulation protocol

The only difference from the dispersed protocol is that each selected dendrite received a group (of size Sclu = 10, 15, 20, 30, and 60) of synapses as opposed to a single synapse. For example, for Nsyn = 60 and Sclu = 20, a total of three dendrites were randomly selected to receive 20 synapses each. We followed the same experimental design as in the dispersed protocol. Thus, for a given number of synapses Nsyn, at each allocation step, one dendrite was chosen at random and Nsyn synapses were placed at random locations within this particular dendrite. For the selected dendrite, synaptic location was randomly changed five times. This process was repeated *N* times, where *N* was the number of dendrites for each model cell.

### Artificial neural network models

We constructed feedforward, two-layer, artificial neural networks with different activation functions in the hidden layer and a single-unit linear output. The hidden layer received a vector containing the number of activated synapses in each dendrite as input. The output layer was a linear layer with a single output, the predicted spiking frequency. We used four different configurations for the hidden layer nonlinearities: (a) in the linear case, the hidden layer was a linear layer with five units followed by a linear activation function (or a ReLU activation function)  (b), in the supralinear case the hidden layer was a five-unit linear layer followed by the logistic sigmoid activation function (c) in the sublinear case the hidden layer was again a five-unit linear layer followed by the activation function *y* = (x+2)^0.7^-2. (d) The bimodal network contained both supralinear and sublinear activation functions. The hidden layer was split in two linear layers. The first part received the input vector of the supralinear dendrites and had a sigmoid activation function and the second part the input of the sublinear dendrites and had the same sublinear activation function as in (c). Both layers projected to the single-unit output linearly. The network was trained using stochastic gradient descent with backpropagation, and the Adam optimization algorithm for the learning rate. The loss function used was the mean squared error (squared L2 norm) between the network predictions and actual firing rates. In order to maintain correspondence with biophysical reality, we restricted our ANN weight updates to be positive, in line with our biophysical models that had only excitatory inputs. Please note that under these conditions, a ReLU function is equivalent to a positive-only linear transfer function. The simulations were implemented in PyTorch 0.4.0 using Python 3.6.6 and numpy 1.14.5.

For each cell, we constructed an ANN that corresponded to its number of supralinear/sublinear dendritic units. Performance accuracy was estimated according to the correlation between predicted (by the ANN) and actual mean firing rates generated by the biophysical models for a wide range of stimulation protocols.

### ANN training/testing datasets

Input to the four ANNs consisted of the number of synapses located in the modeled dendritic branches and the target output consisted of the mean firing rate generated by the biophysical models in response to synaptic stimulation. In the biophysical model, these synapses were activated with the Dispersed and Grouped protocols described above, as well as five new protocols using the same pattern of repetition trials as described above: (1) Randomly dispersed or grouped activation of synapses (e.g., Nsyn = 2-60) in the entire dendritic trees. (2) Randomly dispersed or grouped synaptic allocation on supralinear dendrites or on sublinear dendrites, respectively. Linear Regression plots shown in Fig. 6 and Supplementary Fig. 10 for Cells 2 and 7 are representatives of all cells, as shown in Fig. 6.

### Linear regression and statistical analysis

We calculated the correlation coefficient (*R*^2^) between actual mean firing rates (Target rates, in Hz) generated from the biophysical models and predicted mean firing rates (Predicted rates, in Hz) generated by the trained ANNs, respectively. Statistical analysis between Target and Predicted firing rates was performed using Student’s *t*-test.

### Canonical microcircuit model

A previously published model network for contextual memory formation/recall in hippocampal neuronal populations was adapted and used here^46^. The network consists of excitatory and inhibitory neurons which are both modeled as two-stage integrators, based on experimental data primarily from the CA1 area of the hippocampus. Neurons are modeled as two-layer units, consisting of a somatic spiking unit and independent dendritic subunits (20 units for excitatory neurons, ten units for interneurons) capable of nonlinear synaptic integration, dendritic spike initiation and compartmentalized plasticity. Each dendritic subunit integrates the incoming synaptic inputs which reside on it independently, as follows:

5 $$\tau _{\mathrm{b}}\frac{{dV_{\mathrm{b}}}}{{dt}} = \mathop {\sum}\limits_{i,j} {w_jE_{{\mathrm{syn}}}\delta \left( {t - t_{i,j}} \right)-V_{\mathrm{b}}}$$

where *V*_b_ is the dendritic depolarization, *τ*_b_*, E*_syn_ are constants (Model parameters and constants are listed in Supplementary Table 1), *w*_*j*_ is the weight of synapse j and *t*_*i,j*_ are the timings of incoming spikes. Somatic spiking is given by an integrate and fire model with adaptation:

6 $$C\frac{{dV}}{{dt}} = - g_{\mathrm{L}}\left( {V - E_{\mathrm{L}}} \right) - g_{{\mathrm{AHP}}}\left( {V - E_{\mathrm{K}}} \right) + I_{{\mathrm{syn}}}(t)$$

7 $$\tau _{{\mathrm{AHP}}}\frac{{dg_{{\mathrm{AHP}}}}}{{dt}} = a_{{\mathrm{AHP}}}\delta (t - t_{{\mathrm{spike}}}) - g_{{\mathrm{AHP}}},$$

where *V* is the somatic voltage, *C* is the somatic membrane capacitance, *g*_L_, the leak conductance, *E*_L_ the resting potential (0 mV), *g*_AHP_ is the conductance of the adaptation (afterhyperpolarization, AHP) current and *E*_K_ is the AHP reversal potential. *τ*_AHP_ is the adaptation time constant, *a*_AHP_, the quantal increase of *g*_AHP_ after a somatic spike which occurs at time *t*_spike_. The synaptic current reaching the soma *I*_syn_ is given by

8 $$I_{{\mathrm{syn}}}(t) = g_{{\mathrm{syn}}}\mathop {\sum}\limits_n {(V_{{\mathrm{b}},n}(t)) - IPSC(t)}$$

where *IPSC(t)* is the inhibitory input that the neuron receives from FS BC interneurons and *g*_syn_ is the dendritic coupling constant. Somatic spiking occurs when the somatic voltage reaches the spike threshold *θ*_soma_. The backpropagating action potential is modeled by a depolarization component *V*_bAP_ which is added to the depolarization of all the dendritic subunits. *V*_bAP_ *(t)*:

9 $$V_{{\mathrm{bAP}}}\left( t \right) = E_{{\mathrm{bAP}}}e^{ - \frac{t}{{\tau _{{\mathrm{bAP}}}}}}$$

*E*_bAP_ is the peak of the backpropagating depolarization and *τ*_bAP_ is the time constant of the *bAP*. The voltage response function for sublinear dendrites as a function of total excitatory depolarization *E*_syn_ was given by the power-law function *V* *=* *(E*_syn_*)*^0.7^.

Calcium influx Δ*C*_*syn*_ in synapse after a presynaptic spike is dependent on the depolarization of the dendritic subunit using a sigmoid rule that mimics the voltage dependence of the NMDA receptors as follows:

10 $$\Delta C_{{\mathrm{syn}}} = a_{{\mathrm{Ca}}}\frac{1}{{1 + exp\left( { - \frac{{V - 30\;mV}}{{5\;mV}}} \right)}}$$

where *α*_Ca_ is the maximum Ca^+2^ influx and

11 $$V = V_b + V_{bAP}$$

Synapses are initially allocated randomly in dendritic subunits, given initial weight *w*_init_. Calcium influx in a synapse during stimulation is the determinant of plasticity following the synaptic tagging and capture model^46^: Low-to-intermediate levels of calcium after stimulus presentation lead to the generation of a de-potentiation synaptic tag while high levels of calcium lead to a potentiation tag (see Supplementary Table 7). The consolidation of synaptic tags into the weight of synapses is dependent on the level of Plasticity-Related-Proteins (PRPs). We assume somatic PRP synthesis in the version of the model used here. The level of PRPs is increased to its maximum value (1.0) when the total calcium level exceeds the threshold *Θ*_PRP_ and decays exponentially with time constant *τ*_PRP_. The sum of available proteins determines the rate of consolidation of synaptic tags into the permanent weights *w* of synapses. Synaptic weights are subject to homeostatic plasticity, which normalizes the total synaptic input to a neuron over long time scales:

12 $$\frac{{dw_j}}{{dt}} = \frac{1}{{\tau _{\mathrm{H}}}}\left( {1-\frac{{\mathop {\sum}\nolimits_j {w_j} }}{{w_{{\mathrm{init}}}N_{{\mathrm{syn}}}}}} \right)$$

where *w*_init_ is the initial synapse weight of synapses, *N*_syn_ the total number of synapses in the neuron and *τ*_H_ the time constant of homeostatic synaptic scaling.

We adapted the previously published model as follows:

- Interneurons were modeled with ten dendritic subunits
- Interneuron dendrites can be either sublinear, supralinear, linear, or an equal mix of supralinear and sublinear
- The interneuron population was divided in 50% Fast spiking interneurons and 50% SOM interneurons.
- SOM interneuron dendrites were typically sublinear (although linear/nonlinear ones were also tested), while we varied the nonlinearity of dendrites in FS BC cells.

Simulation of memory engram formation takes place according to the following protocol: For each memory being encoded, the afferents which represent the memory are divided in two, same-sized subpopulations: the conditioned (analogous to context) and the unconditioned (analogous to shock) populations. For encoding, these populations are synchronously activated while simulating detailed voltage dynamics. After each encoding, an interstimulus interval is simulated that varies in duration. After memories are encoded, another inter-stimulus interval of 1 day is applied to simulate the effects of homeostasis. The memory engram properties are then assessed by presenting the conditioned stimulus only, and measuring the response of the entire neuronal population. The excitatory neurons with firing rates >10 Hz during recall are considered part of the engram. The sparsity of the response to each memory was evaluated using the Treves–Rolls sparsity metric^62^.

In the first set of simulations one memory was encoded in the neuronal population via the activation of the memory-encoding presynaptic inputs. In the second set of simulations, two memories were encoded in the same population with 1 or 24 h interstimulus interval between them. After encoding, the memories were reactivated and the firing rates of the excitatory population were used to assess the overlap of their engrams.

The connectivity parameters of the model were calibrated following Bezaire et al.^63^ under the constraint that ∼25–30% of the excitatory population participates in the memory engram. The distribution of incoming synapses to FS BC dendrites was either dispersed (synapses allocated uniformly randomly to all dendritic subunits) or grouped (uniformly allocated to ~33% of dendrites). During the control simulations, SOM+ dendrites were sublinear and BS dendrites were bimodal. Additional results were obtained for supralinear, linear, and bimodal SOM+ dendrites, shown in Supplementary Fig. 11.

### Plasticity rules

In summary the model implements four types of plasticity rules:

1. Calcium dependent LTP/LTD with somatic protein synthesis. LTP/LTD is induced and consolidated based on the synaptic tagging and capture model^64,65^.
2. Voltage-dependent calcium influx which simulates the activation of NMDARs, including NMDA-dependent spike plateaus triggered when the dendritic voltage exceeds the dendritic spike threshold^66^.
3. Plasticity of intrinsic neuronal excitability, amounting to a learning-induced increase in the somatic excitability of pyramidal neurons^67,68^, largely attributed to the CREB transcription factor^68^. We simulate the increased excitability through the transient reduction of the AHP current in the neurons in which somatic PRP synthesis is triggered for ∼12 h after the learning event.
4. Homeostatic plasticity: The effect of homeostasis on synaptic weights is modeled using the synaptic weight scaling rule (Turrigiano 2008)^69^ during a simulated period of 24 h after learning. According to this rule, the total synaptic weight of a model neuron remains constant.

The parameters and constants used in the network model are listed in Supplementary Table 7. The network model was written in C++ and run on the high performance computer cluster of the Poirazi lab. Data analysis was done with Python library numpy.

### Reporting summary

Further information on research design is available in the Nature Research Reporting Summary linked to this article.

## Supplementary information

Supplementary Information

Peer Review File

Reporting Summary

## Data Availability

Simulations were performed on a High-Performance Computing Cluster equipped with 312 CPU cores and 1.150 Gigabytes of RAM, under 64-bit CentOS 6.7 Linux. The source code and the data are publicly available in ModelDB (URL: http://senselab.med.yale.edu/ModelDB/showModel.cshtml?model=237595).
