# Supplementary material for: Challenging the point neuron dogma: FS basket cells as 2-stage nonlinear integrators
Source: Nat Commun. 2019 Aug 14;10:3664. doi: 10.1038/s41467-019-11537-7 (PMC6694133; doi:10.1038/s41467-019-11537-7)
Supplement: Supplementary file 1 — Supplementary Information [file 41467_2019_11537_MOESM1_ESM.pdf]

Challenging the point neuron dogma: FS basket cells as 2-stage nonlinear integrators. Tzilivaki et al., Nat. Comm., 2019

Supplementary information

Supplementary Tables:

Supplementary Table 1. Passive properties of biophysical models				
	Soma	*Axon*	*Proximal dendrites*	*Distal Dendrites*
Leak conductance (*g_pas*) [1]	1.315e-4 S/cm^2	3.55e-6 S/cm^2	1.315e-4 S/cm^2	1.34e-5 S/cm^2
Resting Membrane Potential (e_pas) [1,2]	-68 mV	-68 mV	-68 mV	-68 mV
Membrane capacitance (*cm*)[1]	1.2 uf/cm $^{-2}$	1.2 uf/cm $^{-2}$	1.2 uf/cm $^{-2}$	1.2 uf/cm $^{-2}$
Axial Resistance (*Ra*) [1]	172 ohm cm	172 ohm cm	142 ohm cm	142 ohm cm

**Supplementary Table 2. Active properties of biophysical models.**				
Ion channel (S/cm^2)	*Soma*	*Axon*	*Proximal dendrites*	*Distal Dendrites*
Na_v [3,4]	0.145(PFC1-3)/0.396 (Hipp1-3)/0.828(Hipp4,5)	0.675(PFC1-3)/1.296(Hipp1-3)/1.512(PFC4,5)	0.018	0.014
H_v [4]	0.00001	X	x	X
Kdr_v [3] [4]	0.036 (PFC)/0.0432(Hipp)	0.108 (PFC)/0.144 (Hipp)	0.0009	0.009
$Kslow_v$ [4]	0.000725	X	x	X
Kct_v [4]	0.0001	X	x	X
Kca_v [4]	0.02	X	x	X
Ka_v (proximal [5]) [4]	0.0032	X	0.001	0.0009
Ka_v (distal) [5] [4]	x	X	x	0.00216
Cal_v [4]	x	X	0.00003	0.00003
Can_v [4]	x	X	0.00003	0.00003
Cat_v [4]	x	X	0.0002	0.0002
Calcium buffering [4]dynamics	Yes	No	Yes	Yes

Supplementary Table 2: Active membrane conductances across somatic, axonal, proximal (<=100 microns from the soma) and distal (>100 microns from the soma) dendritic compartments. Sodium current conductances are larger in axonal than somatic compartments. Dendritic sodium conductances are ~10 times smaller than axo-somatic conductances.

Supplementary Table 3. Synaptic mechanisms of biophysical models	
Synaptic Current	Conductance Weight
Autaptic GABAa [2] [6]	5.1*e-4*14
Ca permeable AMPA [2,7]	7,5*e-4
NMDA [8]	3.2*e-4*5

Supplementary Table 3: Validated Synaptic conductance weight values of Autaptic GABAa Calcium permeable AMPA and NMDA currents, used in all simulations

Supplementary Table 4. Electrophysiological properties of biophysical models		
Value	*Model*	*Experimental data*
rHeobase *(pA)* [9,10]	150.0 ± 30.0	123± 58
Input Resistance (Ohm) [9,10]	97.7 ± 30.0	182± 83
Spike threshold (mV) [9,10]	-37.0 ± 3.0	-34± 2
Spike amplitude (mV) [9,10]	52.0 ± 2.0	53.0± 8.0
Spike half width (msec) [8,11,12]	0.5 ± 0.1	0.4-0.5/0.3-0.9/ 1.00±0.07
f-i slope (Hz/pA) [2]	0.26 ± 0.03	0.2± 0.002
AHP (mV) [2]	24.6 ± 2.6	24.3 ± 0.7/23± 5

Supplementary Table 4: Validation of electrophysiological properties.

Supplementary Table 5.		
Cell ID	Number of supralinear dendrites	Number of Sublinear dendrites
Hipp 1	162	56
Hipp 2	13	38
Hipp 3	10	40
Hipp 4	90	97
Hipp 5	27	32
PFC 1	34	7
PFC 2	48	5
PFC 3	43	14

Supplementary Table 5: Number of supralinear and sublinear dendrites in each model cell.

**Supplementary Table 6:** Biophysical and connectivity parameters of the canonical microcircuit model [13] [14]		
N_{pyr}	Number of excitatory neurons	400
N_{inh}	Number of inhibitory neurons	50 SOM+<br>50 PV+
$N_{branches}$	Number of dendritic subunits per neuron	20 for excitatory<br>10 for interneurons
$N_{pyr\rightarrow FSBC}$	Total number of synapses from excitatory neurons to FSBC+ interneurons	1000
$N_{pyr\rightarrow SOM+}$	Total number of synapses from excitatory neurons to SOM+ interneurons	2000
$N_{FSBC\rightarrow pyr}$	Total number of synapses from FSBC interneurons to excitatory neurons	10000
$N_{SOM+\rightarrow pyr}$	Total number of synapses from SOM+ interneurons to excitatory neurons	4000
$N_{input\rightarrow pyr}$	Total number of weak connections from input afferents to pyramidal dendrites per memory	8000
$N_{branches}$	Number of dendritic subunits per neuron	20 for excitatory neurons<br>10 for interneurons
E_{syn}	Maximum unitary EPSPs	4mV for excitatory inputs<br>3mV for inhibitory inputs
E_L	Somatic leakage reversal potential	0 mV
ϑ_{soma}	Baseline voltage threshold for somatic spikes	20mV
ϑ_{dspike}	Voltage threshold for dendritic spike	25mV
g_{syn}	Dendritic coupling constant	20 pS
τ_{dend}	Dendritic time constant	20msec
τ_{soma}	Somatic time constant	30msec
τ_{AHP}	Adaptation time constant of excitatory neurons	180msec
a_{AHP}	Adaptation conductance increase after a spike	0.18nS
E_K	Adaptation current reversal potential	-10 mV

τ_{bAP}	Back propagating action potential time constant	17msec
E_{bAP}	Back propagating action potential max amplitude	30 mV
α_{Ca}	Calcium influx rate	0.1msec^{-1}
synTag (x)	Synaptic tag as a function of Calcium Level *x* (Calcium control model)	$\left(\frac{1.3}{1+exp(-10(10x-3.5.))}\right) - \left(\frac{0.3}{1+exp(-19(10x-2.0))}\right)$
Θ_{PRP}	Calcium level threshold for somatic Plasticity-Related Protein (PRP) synthesis	18.0 (a. u.)
τ_{PRP}	Time constant for PRP decay	60 minutes
τ_{H}	Time constant of homeostatic synaptic scaling	7 days
w_{init}	Initial plastic synapse weight	0.3

**Supplementary Table 7.** Dendritic features of the 8 morphologies of FS BC models. Related to **Figure 4** and **Supplementary Figure 12**								
**Feature**	**Cell_7**	**Cell_6**	**Cell_8**	**Cell_1**	**Cell_4**	**Cell_5**	**Cell_2**	**Cell_3**
**Rsub/sup**	0.104	0.206	0.326	0.528	1.078	1.185	2.923	4.1
**D_{sub} volume (μm3)**	35.57	22.11	46.73	18.35	27.24	42.31	37.05	35.93
**FRclu/dis**	0.68	0.79	0.71	0.16	0.33	0.33	0.39	0.38

- Rsub/sup: ratio of the number of sublinear dendrites over the number of supralinear dendrites.
- Rsub/sup activated: ratio of sub/supra dendrites that actually get activated (with 60 synapses).
- FRclu/dis: ratio of the mean firing rate in response to stimulation of 60 synapses in the clustered (placed in 2 dendrites) versus the dispersed (random placement) allocation. Both protocols were run multiple times as to ensure that all dendrites were activated and mean values were calculated. The ratio indicates the proportion of the mean firing outcomes.
- Dsub: average volume of sublinear dendrites.

Supplementary Figures

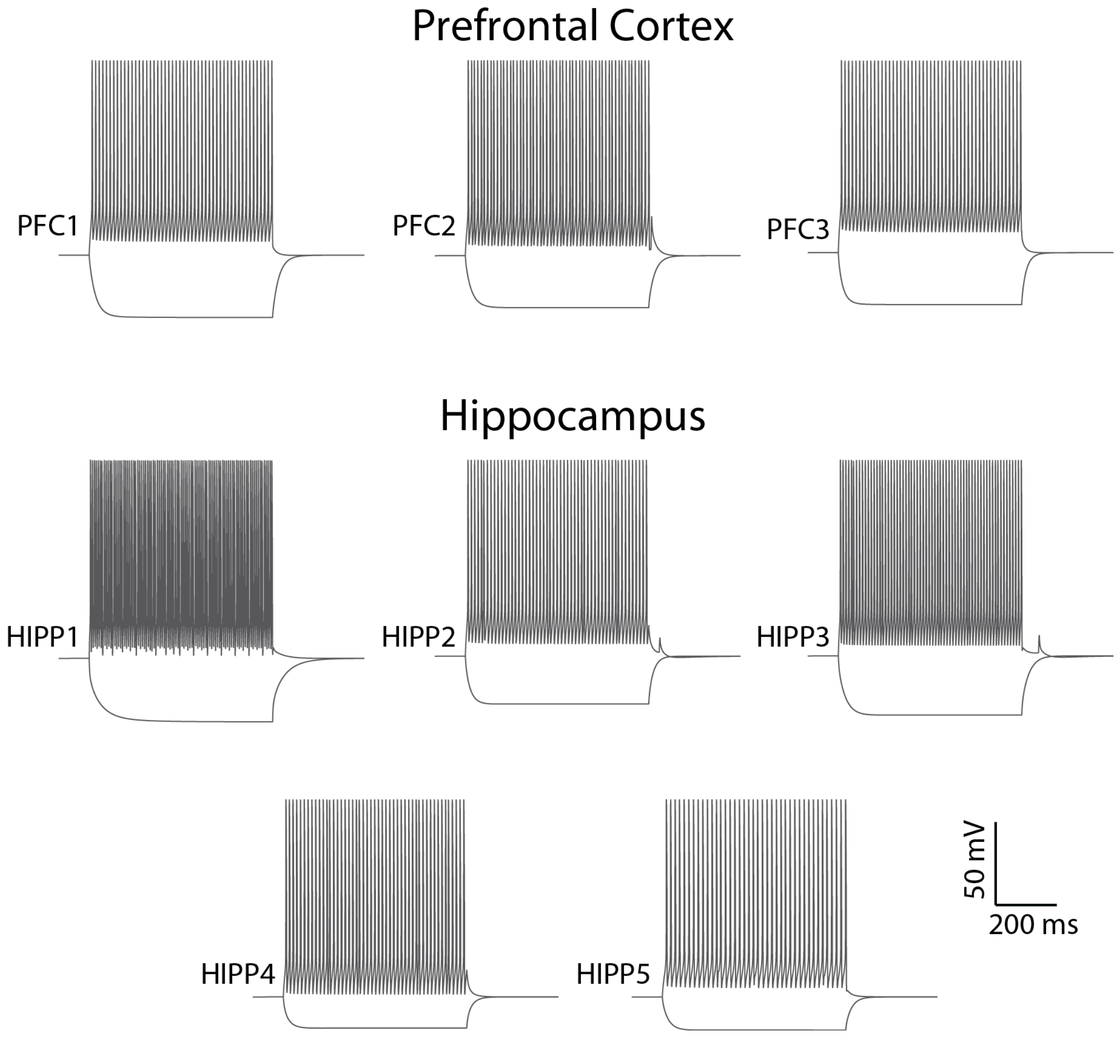

Supplementary Figure 1. Model cell firing profiles. Somatic Current-clamp traces of Hippocampal (A) and PFC (B) model cells, after a depolarizing current injection in somata (500 pA; 1000 ms) evoked a high-frequency firing pattern. A hyperpolarizing current injection in somata (-300pA, 1000ms) induced a realistic hyperpolarizing response.

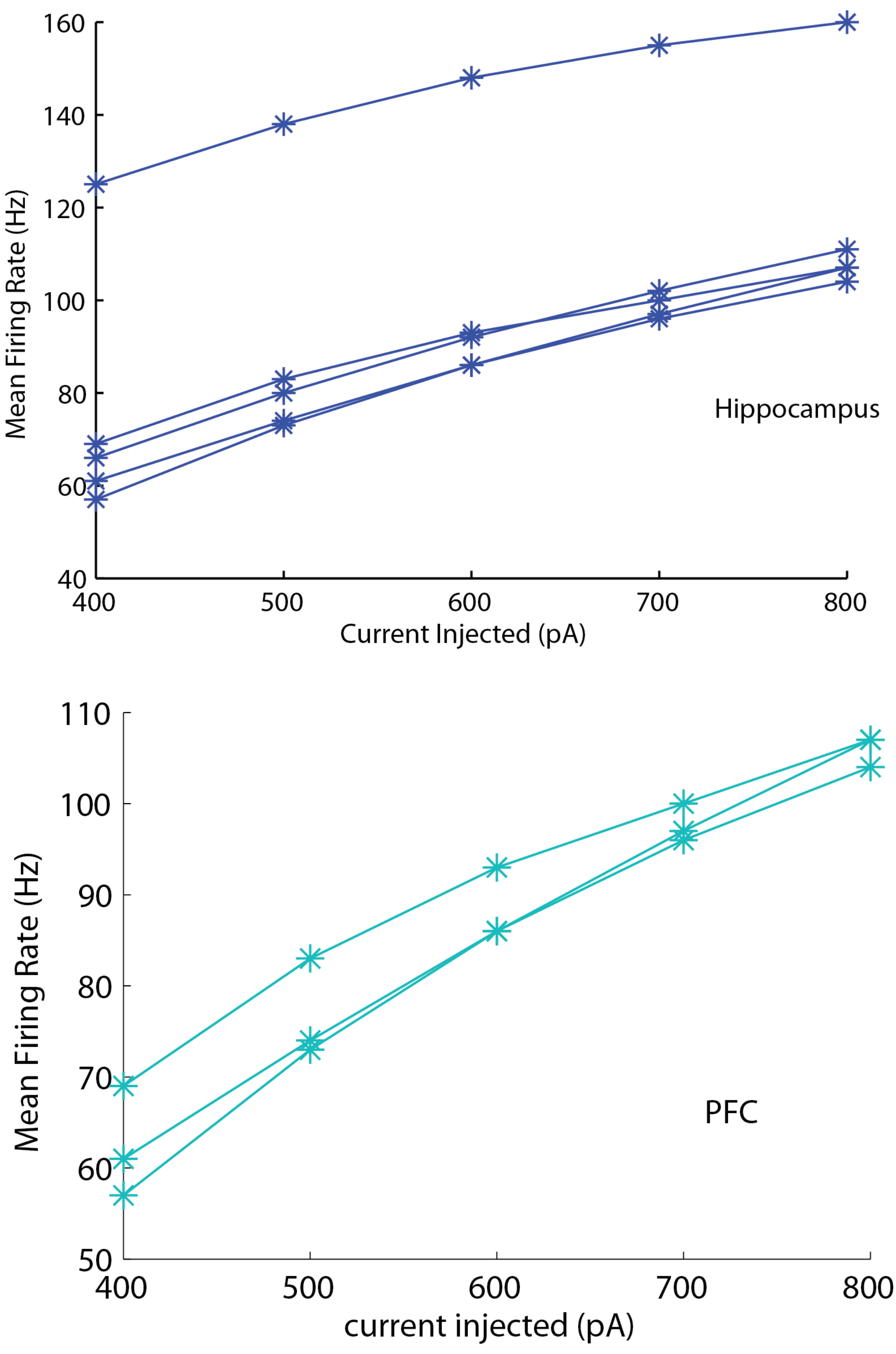

Supplementary Figure 2: Mean firing frequencies in response to injected currents of different amplitudes (600 ms duration) in Hippocampal (up) and PFC (down) model cells.

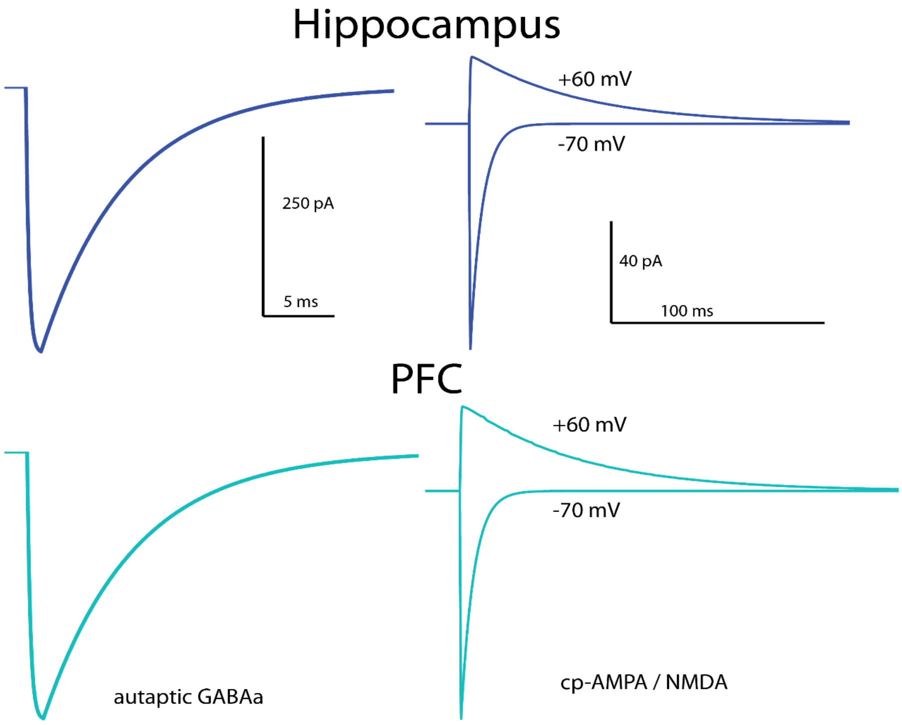

Supplementary Figure 3: Validation of synaptic currents in Fast Spiking basket cells.

Left. A three-step voltage clamp of voltage changes from −70 mV to 10 mV (duration 1 ms) and back to −70 mV was used to produce a self-inhibitory (autaptic) current. During the validation of this current, the reversal potential of Cl− was adjusted from −80 to −16 mV, in order to reproduce the experimental set up of Bacci et al., 2003. However, a physiological reverse potential (−80 mV) was used for all other simulations. Right. Model reproduction of cp-AMPA (−70 mV) and NMDA (+60 mV) currents in response to stimulation of 2 synapses as per Wang et al., 2009. * each trace represents the mean of all Hippocampal and PFC cells respectively.

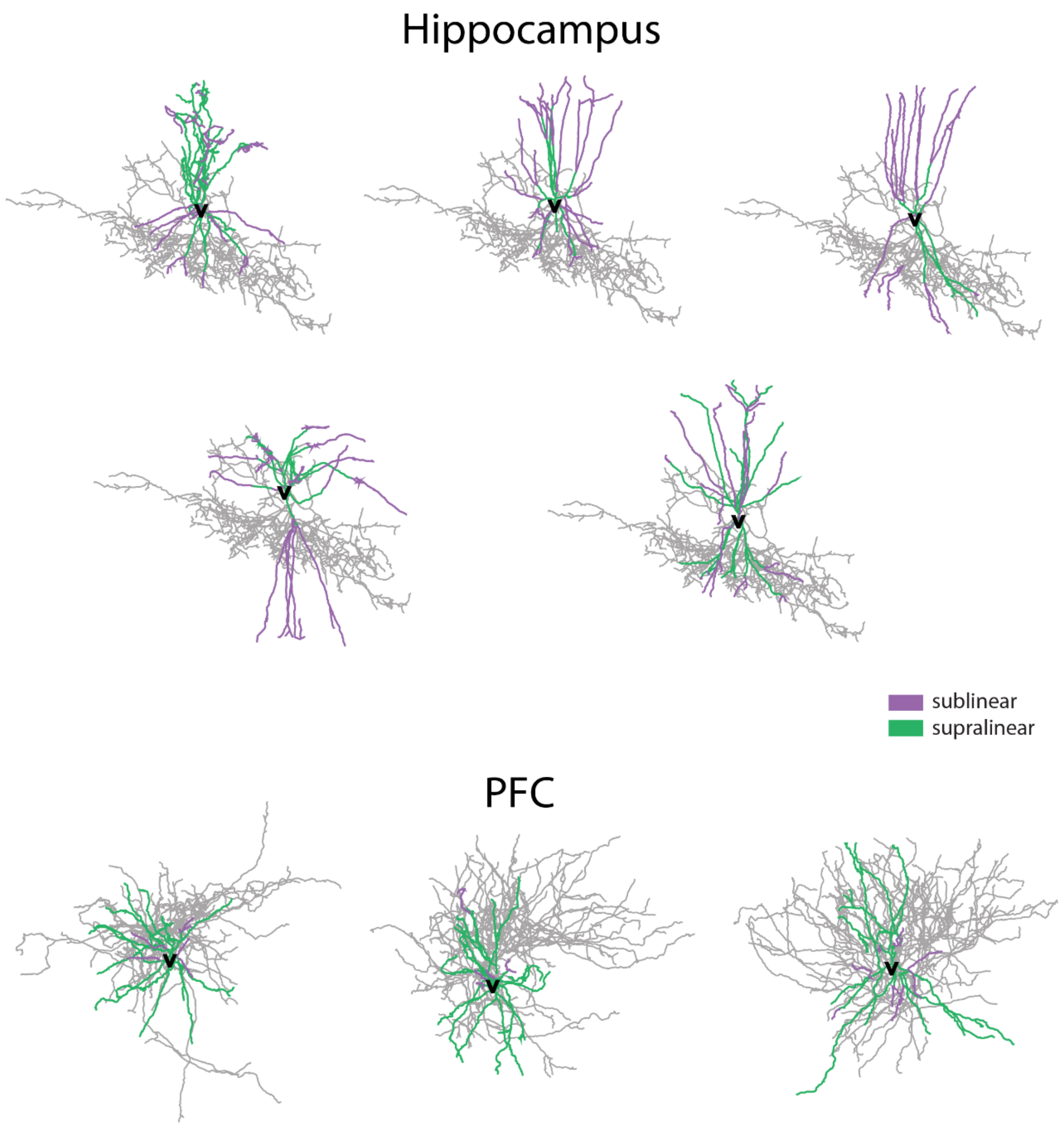

Supplementary Figure 4: Related to **Figure 2**. Bimodal non-linear integration in Fast Spiking basket cells. Supralinear (blue) and sublinear (magenta) dendrites shown in each model cell.

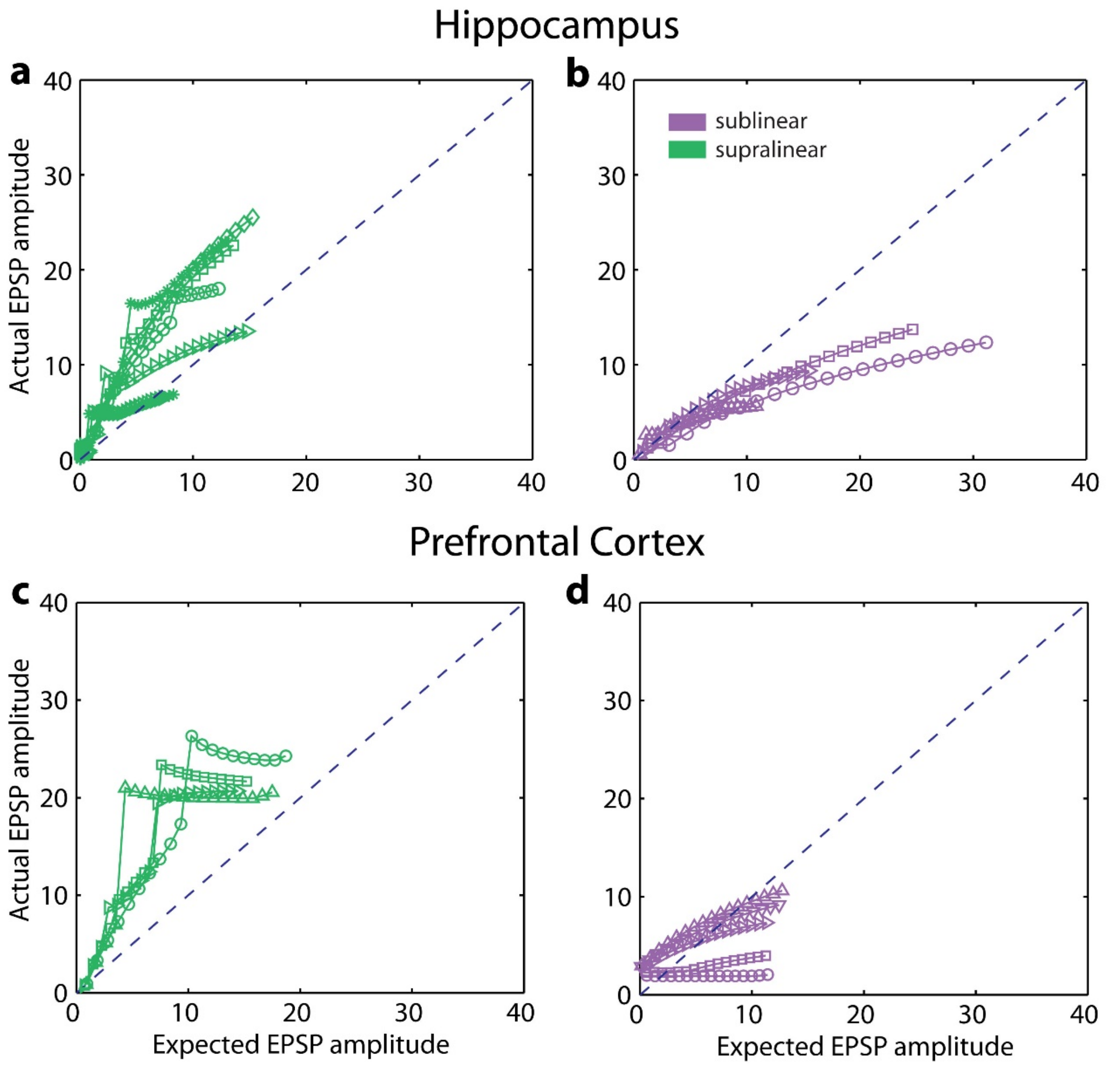

Supplementary Figure 5: Related to **Figure 2**. Bimodal non-linear integration in Fast Spiking basket cells. Representative Somatic EPSPs after stimulation (single pulse) of an increasing number of synapses (1:1:20), uniformly distributed within dendrites.

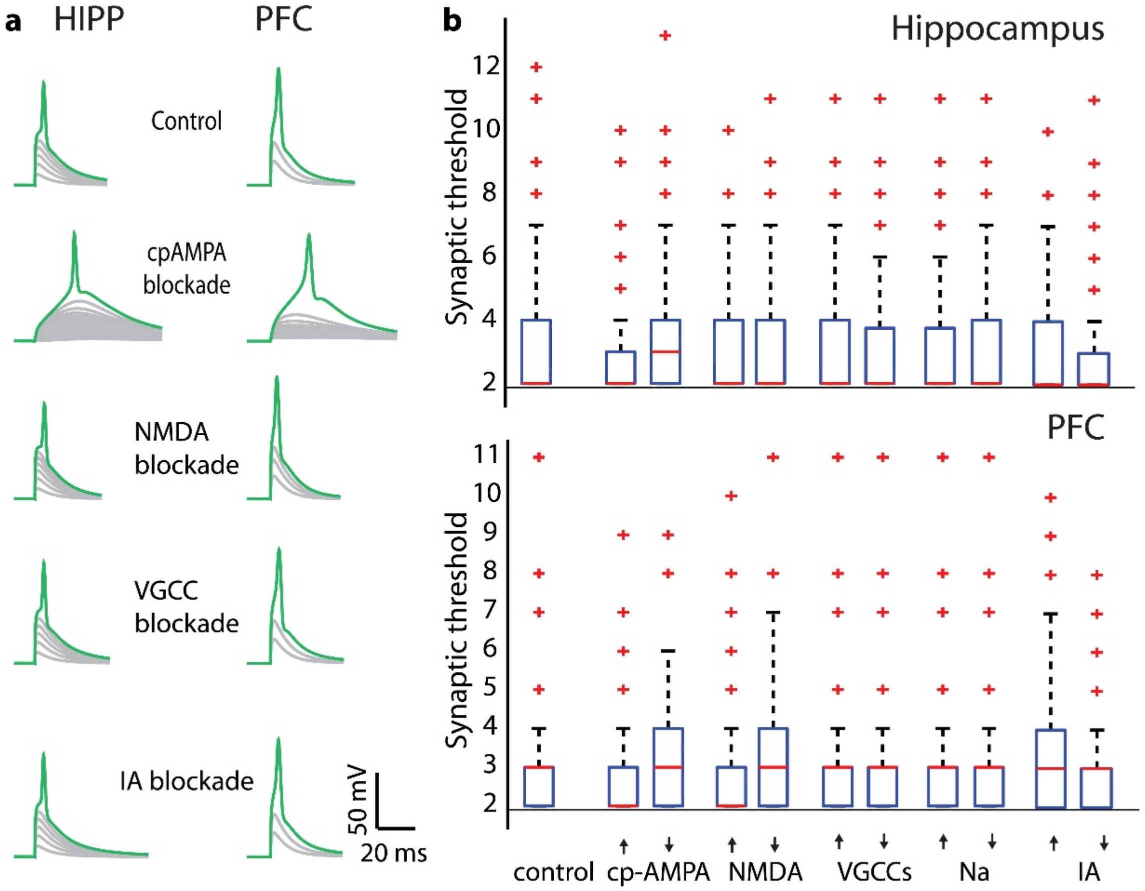

Supplementary Figure 6: **Related to Figure 2**. **a.** Presence of supralinear summation in dendrites of FS BCs after blockade of multiple active currents respectively in Hippocampus (left) and PFC (right). **b.** Sensitivity analysis of biophysical dendritic mechanisms reveals minor changes in the synaptic threshold for spike generation in supralinear dendrites across Hippocampus and PFC. Error bars indicate minimum ans maximum values.

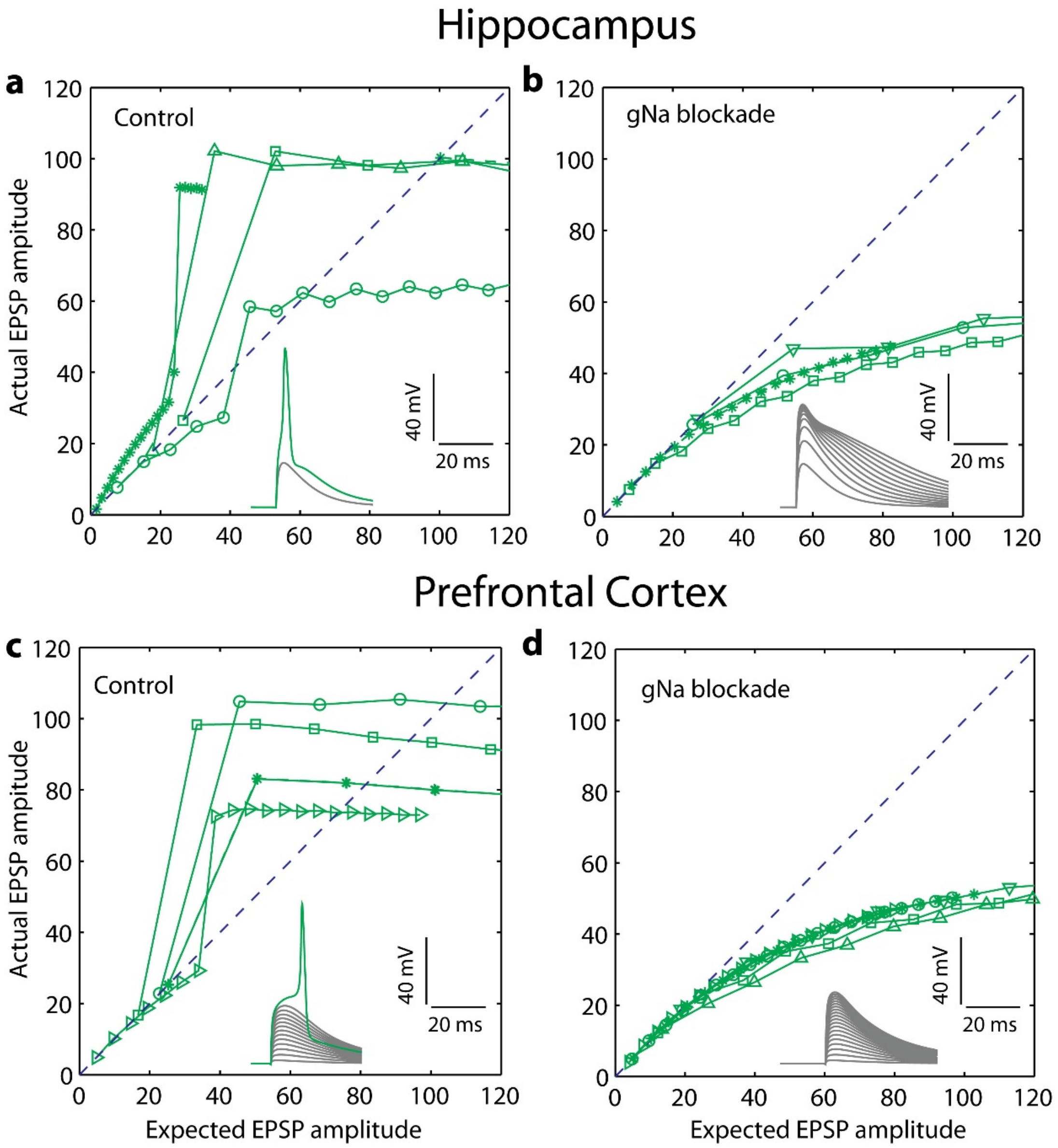

Supplementary Figure 7. Related to **Figure 2**. Blockade of active sodium conductances in the dendrites of FS BCs, totally eliminates the supralinear operation mode. Hippocampal (**a**) and PFC (**c**) representative supralinear responses of dendrites under physiological conditions. Dendritic spikes are eliminated both in Hippocampal (**b**) and PFC (**d**) FS BCs dendrites EPSP responses after blockade of sodium conductances. Linear line represents linear summation.

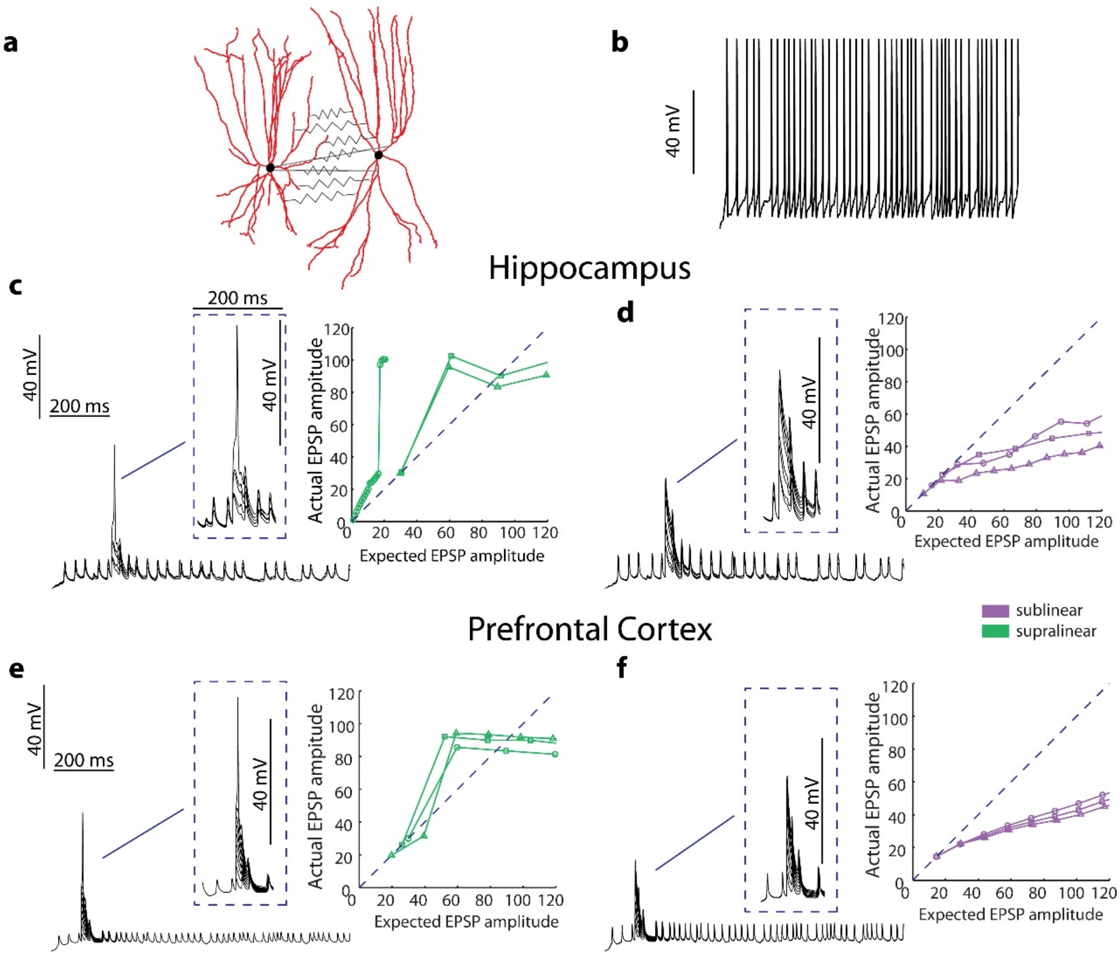

Supplementary Figure 8. FS BCs exhibit supralinear and sublinear dendritic responses in the presence of Gap Junctions. A) Illustrated dendritic trees that are interconnected with Gap Junctions. B) Presynaptic firing rate (~30 Hz). Supralinear (C,E) and sublinear (D,F) dendrites co-exist in Hippocampal (up) and PFC (down) Fast spiking basket cells models.

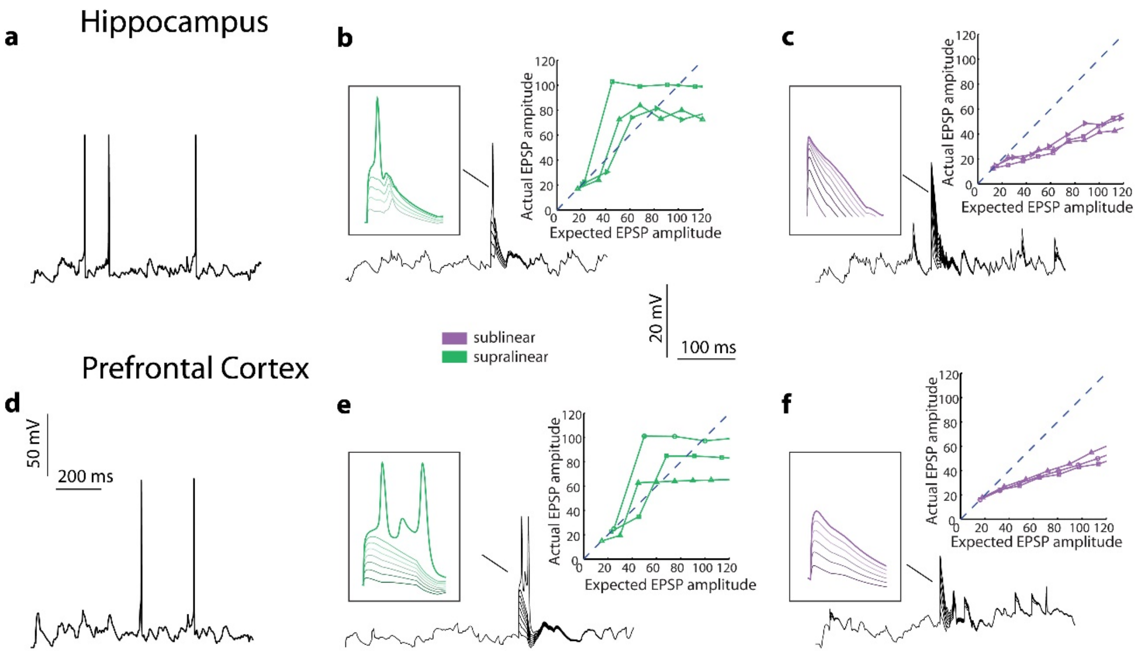

Supplementary Figure 9. FS BCs exhibit supralinear and sublinear dendritic responses in the presence of *in vivo*- like fluctuations. Somatic firing rate of 3±1 Hz induced in Hippocampal (A) and PFC (D) models of FS BCs after synaptic activation of randomly selected dendrites with 10Hz Poisson spike trains. Supralinear (B,E) and sublinear (C,F) dendrites co-exist in FS BCs of Hippocampus (up) and PFC (down).

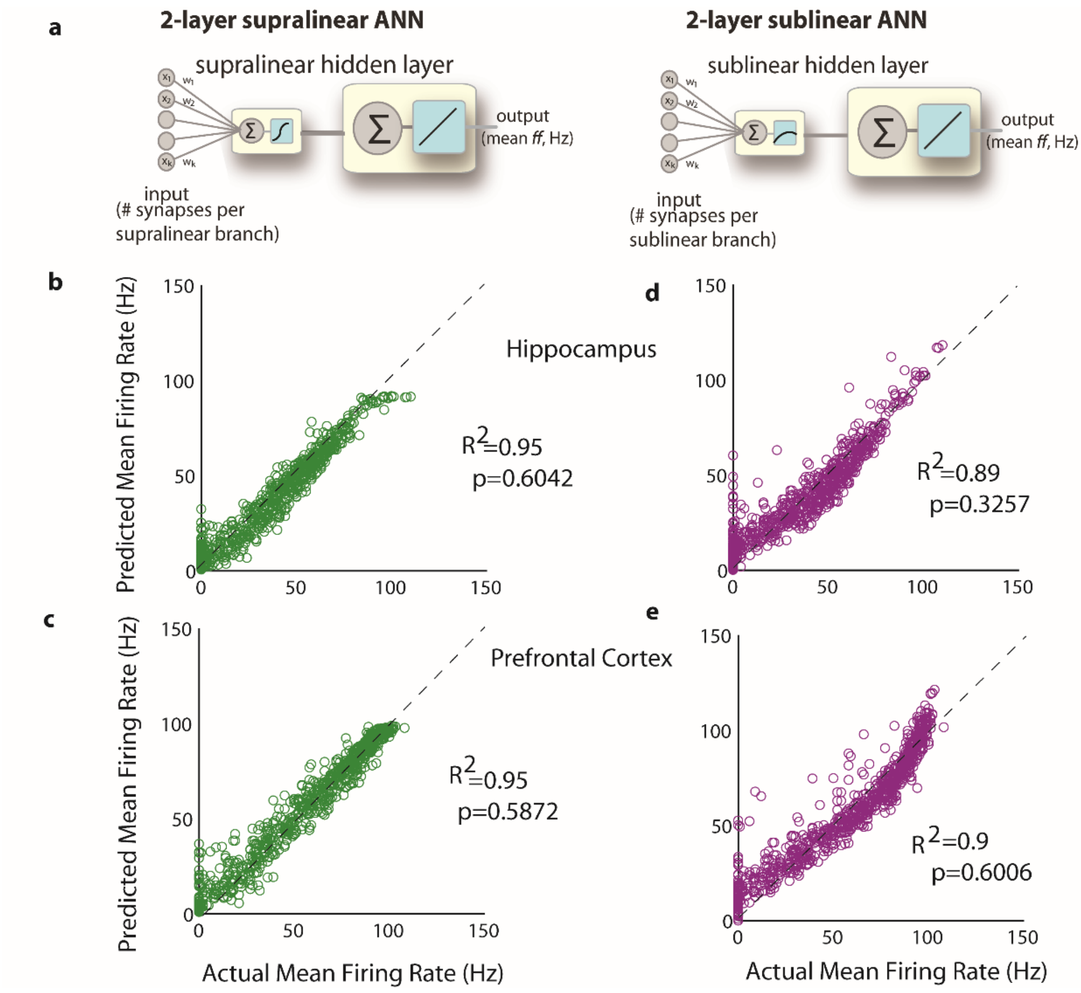

Supplementary Figure 10. Related to **Figure 6**. Linear regression analysis for one hidden layer supralinear (**b**,**c**) and one hidden layer sublinear (**d**,**e**) ANNs for one indicative Hippocampal (top) and one indicative PFC (bottom) model cell. Actual Mean Firing Rates (Hz) correspond to the responses of the compartmental model when stimulating -with 50Hz Poisson spike trains- varying numbers of synapses (1 to 60), distributed in several ways (grouped or dispersed) within both sub- and supra-linear dendrites. Expected Mean Firing Rates (Hz) are those produced by the respective ANN abstraction when receiving the same input (number of stimulated synapses) in its respective sub-/supra- or linear input layer nodes.

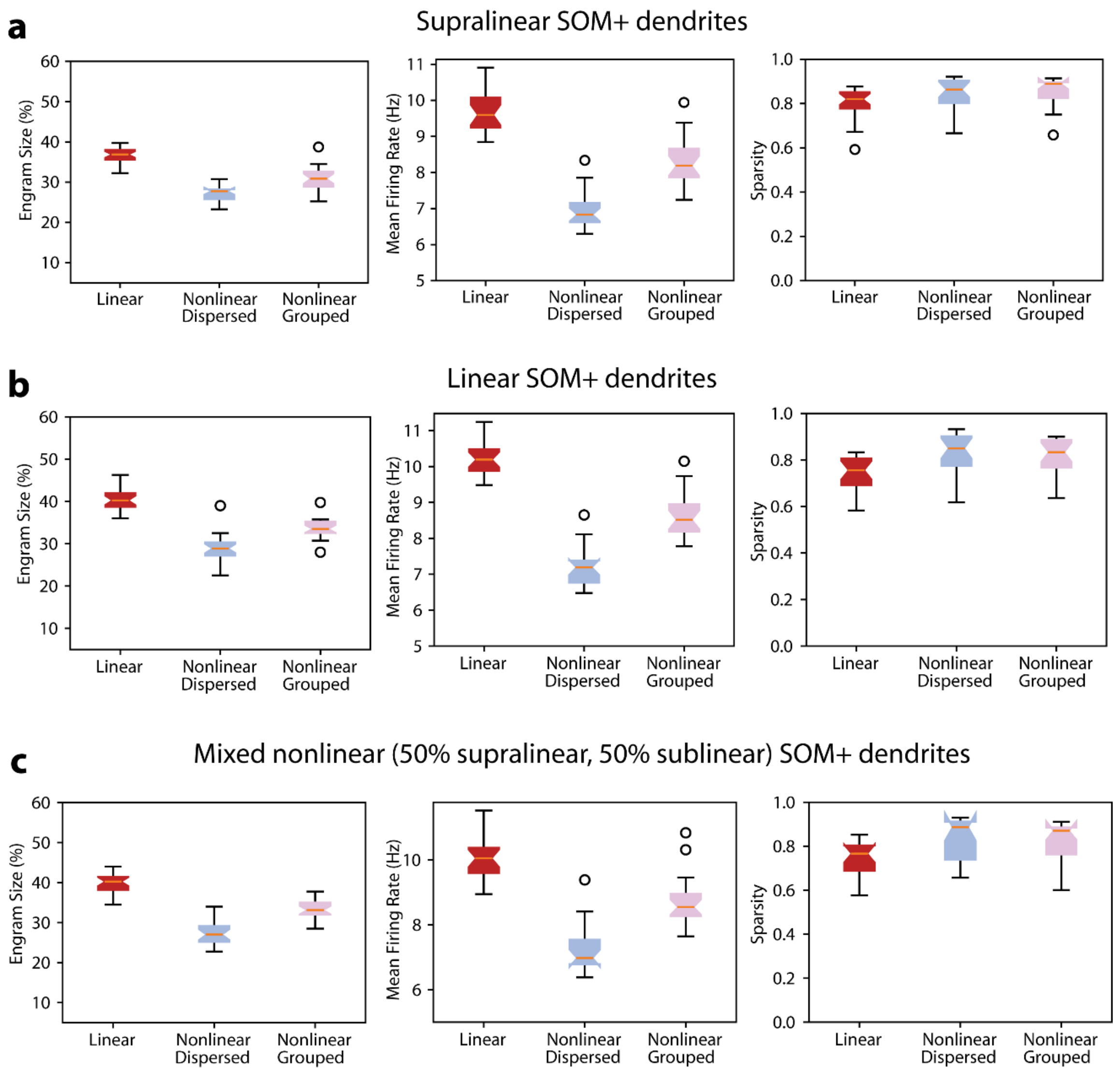

Supplementary Figure 11. Related to **Figure 7.** Manipulation of SOM+ models dendritic transfer function results in almost identical responses of multiple properties of the canonical microcircuit. Modeled SOM+ dendrites A) Supralinear B) Linear C) Supralinear and sublinear (50% of each mode). Error bars indicate minimum and maximum values.

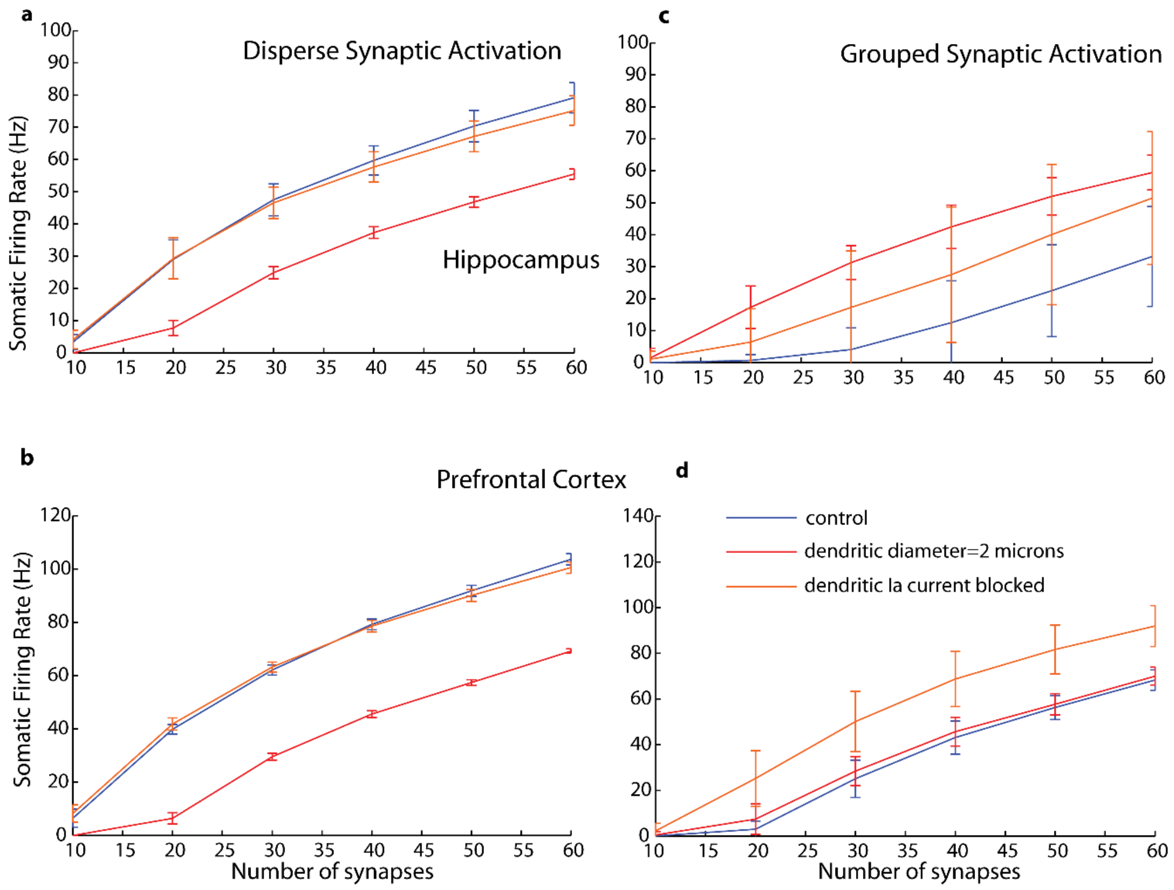

Supplementary Figure 12. Related to **Figure 4:** Firing rate responses (in Hz) from one Hippocampal (**a**, **c**) and one PFC (**b**, **d**) model cell, in response to stimulation of increasing numbers of synapses (10 to 60) that are either randomly distributed throughout the entire dendritic tree or within a few dendritic branches. Effect of dendritic diameter (red, setting the diameter of all dendrites to 2 microns) and A-type current (orange, setting the conductance of dendritic A-type currents to zero) on somatic firing rates in response to synaptic stimulation under dispersed and grouped spatial arrangements. As shown in panels **a**, **c** disperse synaptic arrangements benefit mostly from the dendritic morphology of FS BCs, as setting the diameter to 2 microns sharply decreases this preference. Grouped arrangements on the other hand (panels **b**, **d**) are severely hampered by the high conductance of the A-type potassium channels in these cells, as blockade of these currents enhances somatic output. This potassium current does not penalize disperse inputs as much, simply because it is not as strongly activated as in the case of grouped activation (which induces much higher local depolarizations and thus stronger A-type channel activation). (Student's t-test. p-values for the various comparisons: hippocampus, disperse: diameter vs. control =0.0018, IA vs. control, non-significant; hippocampus, grouped: diameter vs. control=0.0048, IA vs. control=0.0087; PFC, disperse diameter vs. control =0.0014, IA vs. control, non-significant; PFC, grouped diameter vs. control =0.0102, IA vs. control=0.0026).Error bars indicate minimum and maximum values.

Supplementary References

1. Nörenberg, A., Hu, H., Vida, I., Bartos, M. & Jonas, P. Distinct nonuniform cable properties optimize rapid and efficient activation of fast-spiking GABAergic interneurons. *Proc. Natl. Acad. Sci. U. S. A.* **107**, 894–9 (2010).
2. Bacci, A., Rudolph, U., Huguenard, J. R. & Prince, D. A. Cellular/Molecular Major Differences in Inhibitory Synaptic Transmission onto Two Neocortical Interneuron Subclasses.
3. Hu, H., Gan, J. & Jonas, P. Interneurons. Fast-spiking, parvalbumin$^+$ GABAergic interneurons: from cellular design to microcircuit function. *Science* **345**, 1255263 (2014).
4. Konstantoudaki, X., Papoutsi, A., Chalkiadaki, K., Poirazi, P. & Sidiropoulou, K. Modulatory effects of inhibition on persistent activity in a cortical microcircuit model. *Front. Neural Circuits* **8**, (2014).
5. Goldberg, J. H., Yuste, R. & Tamas, G. Ca2+ imaging of mouse neocortical interneurone dendrites: contribution of Ca2+-permeable AMPA and NMDA receptors to subthreshold Ca2+dynamics. *J. Physiol.* **551**, 67–78 (2003).
6. Connelly, W. M. & Lees, G. Modulation and function of the autaptic connections of layer V fast spiking interneurons in the rat neocortex. *J. Physiol.* **588**, 2047–63 (2010).
7. Goldberg, J. H. *et al.* Calcium Microdomains in Aspiny Dendrites. *Neuron* **40**, 807–821 (2003).
8. Wang, H.-X. & Gao, W.-J. Cell type-specific development of NMDA receptors in the interneurons of rat prefrontal cortex. *Neuropsychopharmacology* **34**, 2028–40 (2009).
9. Povysheva, N. V *et al.* Parvalbumin-positive basket interneurons in monkey and rat prefrontal cortex. *J. Neurophysiol.* **100**, 2348–60 (2008).
10. Povysheva, N. V, Zaitsev, A. V, Gonzalez-Burgos, G. & Lewis, D. A. Electrophysiological heterogeneity of fast-spiking interneurons: chandelier versus basket cells. *PLoS One* **8**, e70553 (2013).
11. Liu, Y.-C., Cheng, J.-K. & Lien, C.-C. Rapid Dynamic Changes of Dendritic Inhibition in the Dentate Gyrus by Presynaptic Activity Patterns. *J. Neurosci.* **34**, 1344–1357 (2014).
12. Lazarus, M. S. & Huang, Z. J. Distinct maturation profiles of perisomatic and dendritic targeting GABAergic interneurons in the mouse primary visual cortex during the critical period of ocular dominance plasticity. *J. Neurophysiol.* **106**, 775–787 (2011).
13. Kastellakis, G., Silva, A. J. & Poirazi, P. Linking Memories across Time via Neuronal and Dendritic Overlaps in Model Neurons with Active Dendrites. *Cell Rep.* **17**, 1491–1504 (2016).
14. Bezaire, M. J., Raikov, I., Burk, K., Vyas, D. & Soltesz, I. Interneuronal mechanisms of hippocampal theta oscillations in a full-scale model of the rodent CA1 circuit. *Elife* **5**, 1–106 (2016).
