# Supplementary material for: Challenging the point neuron dogma: FS basket cells as 2-stage nonlinear integrators
Source: Nat Commun. 2019 Aug 14;10:3664. doi: 10.1038/s41467-019-11537-7 (PMC6694133; doi:10.1038/s41467-019-11537-7)
Supplement: Supplementary file 2 — Peer Review File [file 41467_2019_11537_MOESM2_ESM.pdf]

Editorial Note: This manuscript has been previously reviewed at another journal that is not operating a transparent peer review scheme. This document only contains reviewer comments and rebuttal letters for versions considered at Nature Communications .

Reviewers' Comments:

Reviewer #1:
Remarks to the Author:
I have reviewed this paper when it was submitted to [redacted]. The authors addressed all my concerned. The code is much cleaner. I would like to thank the authors for that.
